# Elaborate plumage patterning in a Cretaceous bird

**DOI:** 10.7717/peerj.5831

**Published:** 2018-11-02

**Authors:** Quanguo Li, Julia A. Clarke, Ke-Qin Gao, Jennifer A. Peteya, Matthew D. Shawkey

**Affiliations:** 1 State Key Laboratory of Biogeology and Environmental Geology, China University of Geosciences, Beijing, China; 2 Jackson School of Geosciences, University of Texas at Austin, Austin, TX, USA; 3 School of Earth and Space Sciences, Peking University, Beijing, China; 4 Department of Biology, University of Akron, Akron, OH, USA; 5 Evolution and Optics of Nanostructures Group, Department of Biology, University of Ghent, Belgium

**Keywords:** Color pattern, Diversity, Crypsis, Melanin, *Confuciusornis*

## Abstract

Integumentary patterns and colors can differentiate species, sexes, and life changes and can inform on habitat and ecology. However, they are rarely preserved in the fossil record. Here, we report on an extremely well-preserved specimen of the Cretaceous bird *Confuciusornis* with unprecedented complexity, including small spots on the wings, crest, and throat. Morphological and chemical evidence suggest that these patterns are produced by melanin, but unusual preservation prevents assignment of specific colors. Based on comparisons with extant birds, these patterns were likely used for camouflage, although other functions including sexual signaling cannot be ruled out. Our data show that even more elaborate plumage patterns than the spangles in *Anchiornis* and stripes in *Sinosauropteryx* were present at a relatively early stage of avian evolution, showing the significance of coloration and patterning to feather evolution.

## Introduction

Despite its significance to organismal evolution, color in the vertebrate fossil record was for a long time thought to be essentially unknowable due to the lack of preservation of color-producing pigments and structures (reviewed in Vinther, 2015; D’Alba & Shawkey, 2018). The discovery of organelle (melanosome) remains with the widespread pigment melanin preserved in the fossil record (Vinther et al., 2008) has allowed the reconstruction of melanin-based animal colors (Clarke et al., 2010; Li et al., 2010; Li et al., 2012; Zhang et al., 2010). These studies have shown that feather colors spanned the range of those produced by melanin, including black, brown, gray, and iridescent (Clarke et al., 2010; Li et al., 2010; Li et al., 2012; Zhang et al., 2010). Color patterns similarly ranged from uniform (Li et al., 2012) to countershaded (dark dorsum and light ventrum; Clarke et al., 2010; Vinther et al., 2016) to somewhat more complex, including leg and tail spangles (Li et al., 2010). Taken together, these studies suggest that color and color pattern, whether used for crypsis or sexual signaling, were prevalent and diverse in fossil animals. However, if these plumage patterns reached the levels of complexity of some of those in modern animals is unclear. The spangled patches in *Anchiornis*, for example, showed the presence of within-feather patterning, but not multiple-feather spots such as those in thrushes or nightjars. Identifying such patterns could be important both because of their significance to ecology and to the evolution of developmental pathways of plumage pigmentation (Prum & Williamson, 2002). Here, we report on elaborate plumage patterning of an exceptionally-preserved specimen of *Confuciusornis*.

## Materials and Methods

We examined color pattern, melanosome morphology, and chemistry in a target specimen (CUGB P1401; China University of Geosciences, Beijing). Morphology of melanosomes differs from that of bacteria in consistent ways (reviewed in Vinther, 2015), but several other studies have suggested direct chemical data is needed to verify that the fossilized microbodies are melanosomes (Moyer et al., 2014; Lindgren et al., 2015a). Although all of these studies have identified chemical data consistent with melanin using either trace metal mapping (Wogelius et al., 2011) or comparison of recovered chemical signatures with melanin standards by various techniques (Glass et al., 2012; Lindgren et al., 2014, 2015b; Colleary et al., 2015), some of the same authors have concluded that the identity of these structures as fossil melanosomes is still in doubt (Moyer et al., 2014; Lindgren et al., 2015b; Schweitzer, Lindgren & Moyer, 2015). It is therefore worthwhile to examine these structures in detail using both morphology and chemistry.

**Figure 1:**
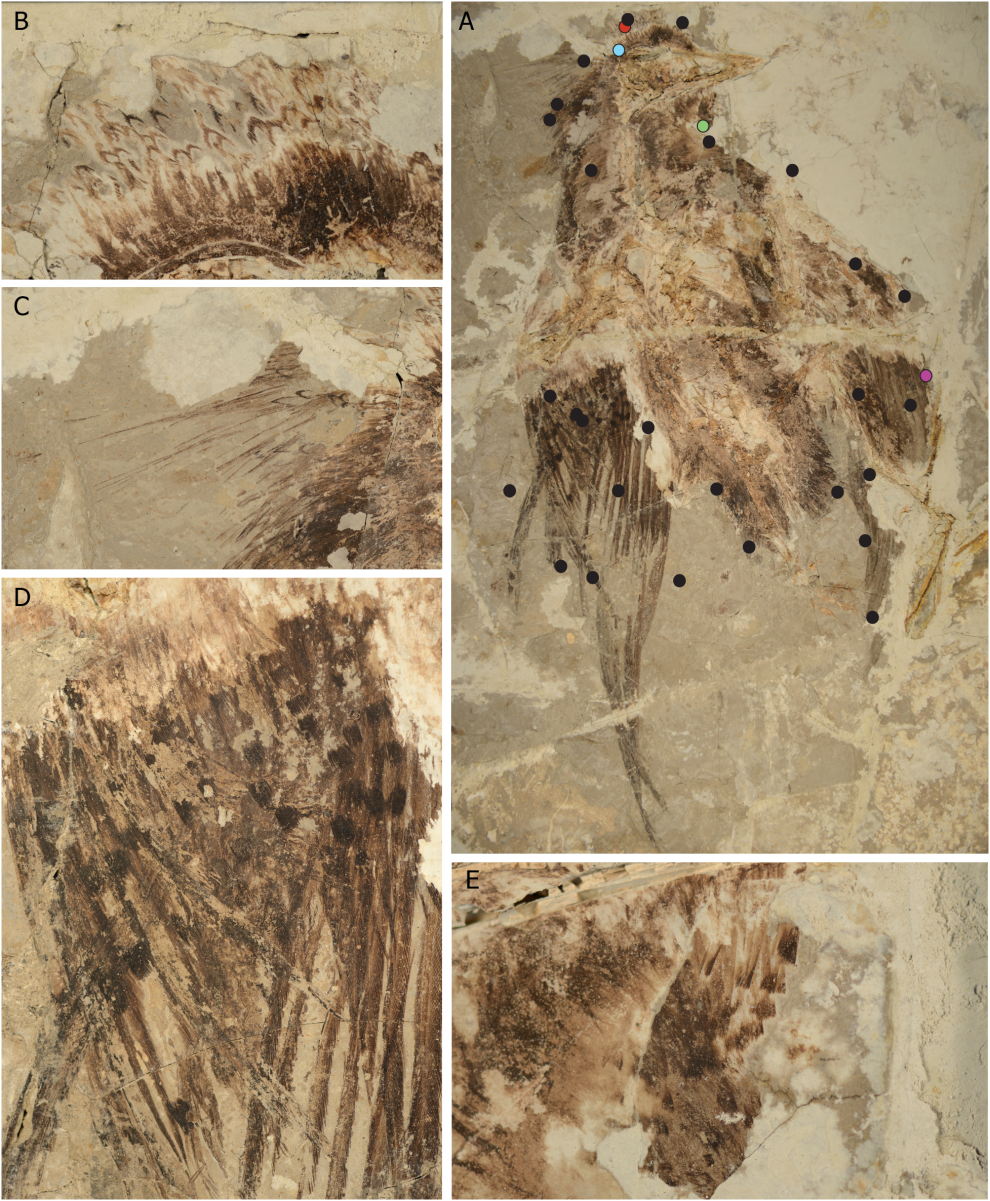
Evidence of plumage diversity in the Confuciusornithidae from the new specimen (CUGB P1401). (A–D) The primary slab of CUGB P1401 showing details of the plumage including crest ornamentation on the (B) top and (C) back of the head as well as on the (D) secondary and (E) gular feathers. Dots indicate locations sampled for Raman and morphological analyses. Colored dots correspond to locations of SEM images and Raman spectra in Fig. 2.

**Figure 2:**
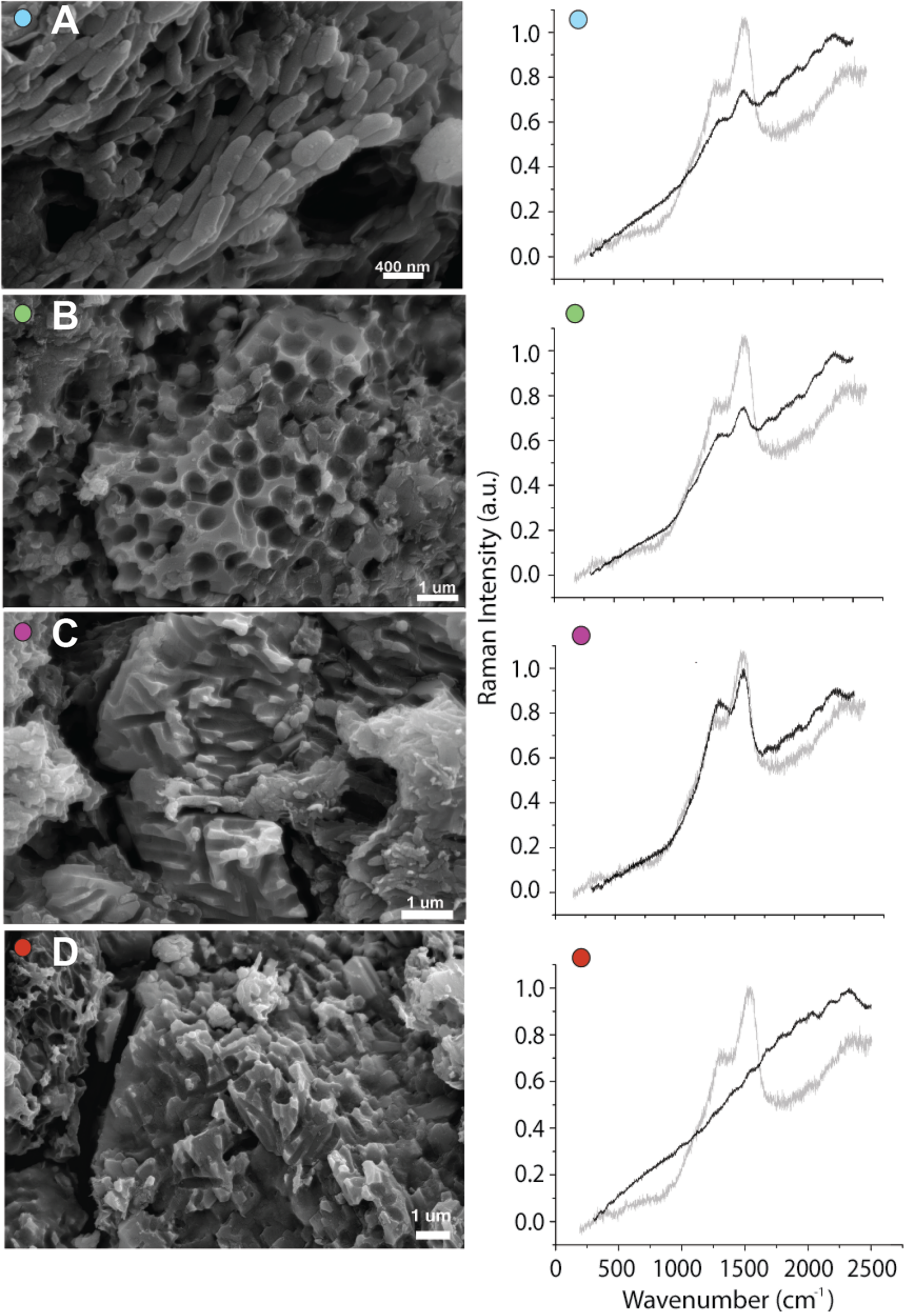
Melanosome diversity in CUGB P1401. Scanning electron microscope images showing representative 3D preservation or impressions of (A) ellipsoid, (B) spherical, (C) elongate, and (D) mixed morphologies of melanosomes in feathers and associated Raman spectra. Raman spectra of all but (D) show possible eumelanin peaks (gray spectrum is each panel is melanin extracted from feathers of an extant Mallard) but these signatures vary in strength. No signature for pheomelanin was recovered from any extant sample, including those known to have high proportions of pheomelanin (Fig. S5). Colored dots correspond to sampling locations on Fig. 1.

Using a beveled probe, we took two 1 mm^2^ samples from each of 32 areas of the plumage and, as negative controls, the matrix (Figs. 1 and 2; Fig. S2; Table S2). One sample was used for morphological analysis with scanning electron microscopy while its matching pair was used for chemical analyses. We rigorously tested these paired samples using multiple standard chemical techniques: confocal Raman spectroscopy, time-of-flight secondary ion mass spectrometry (ToF-SIMS), and matrix-assisted laser desorption/ionization mass spectrometry (MALDI, used here for the first time to examine melanin in fossil samples).

### Raman analyses

For comparison of Raman spectra from extant and fossil samples, we extracted melanin from contour feathers of black chicken *Gallus gallus* and Red-winged Blackbird *Agelaius phoeniceus*, brown Cooper’s Hawk *Accipiter cooperii*, House Wren *Troglodytes aedon* and red chicken (Rhode Island Red rooster, known to have high pheomelanin content (Smyth, Porter & Bohren, 1951), and iridescent Wild Turkey *Meleagris gallopavo* and Mallard *Anas platyrhynchos* using a standard Proteinase-K based method (Liu et al., 2003). We purchased *Sepia* melanin from Sigma (Sigma-Aldrich M2649, Bornem, Belgium) as a eumelanin standard for comparison. To compare Raman spectra of fossils with feather keratin, we obtained a white flight feather from a sulfur-crested cockatoo *Cacatua galerita*. We grew the feather-degrading bacterium *Bacillus licheniformis* (Burtt & Ichida, 1999; kindly provided by Dr. E.H. Burtt, Ohio Wesleyan University) on a white Mallard nape feather that had been sterilized in an autoclave at 120 °C for 20 min. A cluster of bacteria was placed onto the sterilized feather and incubated overnight at 37 °C. Carbon black (Peteya et al., 2017), graphite, and bituminous coal were also analyzed as controls for any carbon present on the surface of the fossil samples. Additionally, two mm sections of carbonized plants were analyzed as controls for carbonization, including one sample from the midrib of a *Platanus* leaf (PTRM #20639) from the Late Cretaceous Hell Creek Formation, Mud Buttes locality (PTRM #P88002), Marmarth, North Dakota, and one sample from the lycopsid root *Stigmaria* (CMNH P-21773) from the Upper Carboniferous Joggins Formation, Joggins, Nova Scotia (Peteya et al., 2017).

Raman data were collected on a LabRAM High Resolution confocal Raman microscope (HORIBA Scientific) with a 532 nm laser through a 50× lens objective. Raman spectra for all samples were generated with an integration time of 5 s × 1 accumulation, a 100 μm slit aperture, a 400 μm pinhole and a grating of 1,200 lines per mm. Raman spectra were collected from dark areas on each sample that correlated with preserved feathers. Spectra from matrix samples were collected as negative controls from areas that appeared representative (e.g., not from small aggregations of minerals that made up a very small percentage of the sample). Fossil and matrix samples were irradiated with a D1 (10%) filter for 10 min to reduce fluorescent signals before spectra were collected with a 50× objective. Data were collected sequentially using D1, D0.6 (25%), and D0.3 (50%) filters to test repeatability of spectra. Cosmic ray spikes were removed and samples were checked for burning between each filter trial. This process was also repeated on at least one other spot on the sample to test repeatability.

Extant melanin, keratin, carbon controls, and bacteria were not initially irradiated to avoid burning the samples. Spectra were collected sequentially using D1, D0.6, and D0.3 filters for modern feather melanin samples. Spectra for Mallard melanin were collected using a D2 (1%) filter because the sample was easily burned. This procedure was repeated at least once on another part of the sample. For the keratin sample, no filter was used and spectra were collected from at least two areas on both the external surface of the rachis and the barbs. For the bacterial sample, Raman spectra were generated from the center of the cluster using D2, D3 (0.1%), and D4 (0.01%) filters. For all extant samples, cosmic ray spikes were removed and the sample was checked for burning between each filter trial. If the sample was burned, the spectra were deleted and the protocol begun from a new spot. All spectra were normalized in Origin Pro (OriginLab, Northampton, MA, USA) and peak fitting was conducted for each sample using a Gaussian distribution with a linear baseline in IgorPro (Wavemetrics, Lake Oswego, OR, USA).

### Time-of-flight secondary ion mass spectrometry

We used ToF-SIMS as a corroborative technique for detecting melanin in the *Confuciusornis* samples. ToF-SIMS analyses were performed using a TOF.SIMS 5-100, ION-TOF GmbH at the Analysis Center of Tsinghua University in the static SIMS using 30 keV Bi3^+^. The scanning area was 10–100 × 10–100 μm and the diameter of the beam spot of the primary ion was five μm. We compared four of the fossil feather samples (k1, o, p, y) to a matrix sample and three extant feather samples, including chicken, turkey, and crow feathers. Mass spectra were taken at 65 × 65 μm resolution for all samples. We based the masses of our ToF-SIMS data on the theoretical masses for melanin compounds presented by Lindgren et al. (2015a) and calculated the relative intensity of spectra for CUGB P1401 and extant samples at those theoretical masses. Relative intensities were calculated by dividing the observed intensity by the sum of the intensities of all peaks at each observed mass (Lindgren et al., 2015a), then mean-centered to the relative intensities for all peaks at that particular mass using the Standardize function in Excel. The standardized intensities were then compared using principal component analysis in R.

### Matrix-assisted laser desorption/ionization mass spectrometry

We further tested the chemistry of the fossil samples using MALDI on a Bruker Ultraflex III MALDI tandem time-of-flight (ToF/ToF) mass spectrometer (Bruker Daltonics, Billerica, MA, USA) at The University of Akron. MALDI has not previously been employed for the chemical characterization of melanins in the fossil record. A total of 12 CUGB P1401 samples (a1, b1, d1, e1, f1, k1, t, u, v, w, y, z) were individually mixed with a 0.1M ammonium acetate buffer to extract any melanin present in the sample. No matrix was used to absorb excess laser energy in order to avoid interference. Samples remained in the buffer solution for approximately 24 h, then the resultant supernatant was applied to a MALDI sample target and allowed to dry at room temperature. We collected the spectra using a 355 nm Nd:YAG laser in reflection mode, with ion source 1 (IS1) at 25.03 kV, ion source 2 (IS2) at 21.72 kV, source lens at 9.65 kV, reflectron 1 at 26.32 kV, and reflectron 2 at 13.73 kV. Fossil spectra were compared to a series of carbon controls, including graphite, bituminous coal, carbon black, and plant fossil samples (*Stigmaria* CMNH P-21773 and *Platanus* PTRM #20639), melanin extracted from black feathers of the Red-winged Blackbird (*Agelaius phoeniceus*), and a fossil test sample. The fossil test sample was removed from the primary wing feathers of an undescribed dinosaur from the Jurassic Yanliao Biota and is known to have preserved melanosomes. All additional spectra were collected using the same parameters as the CUGB P1401 samples.

## Results

The specimen, preserved in part (Slab A) and counterpart (Slab B), exhibits exceptional feather preservation but poor preservation of bone (Figs. 1 and 2; Figs. S1 and S2; Table S1). It was recovered from Early Cretaceous deposits in Fengning County, Hebei Province, with the beds estimated to be equivalent with the Dawangzhangzi Member of the Yixian Formation (Gao, Chen & Jia, 2013). The specimen is referable to the Confuciusornithidae based on preserved autapomorphies of this clade (see Supplement) and is differentiated from all described species except for *Confuciusornis sanctus* (Table S1). Feathers on the dorsal surface of the skull anterior to the frontal/premaxillae contact are tiny, narrow and darken distally. These feathers are especially well preserved in Slab A. While in Slab B only dark tips can be observed, in Slab A, the dark region of the feather tip is discernibly a thin dark, recurved distal-most band (Fig. 1B; Fig. S1). Feathers of this morphology lengthen toward the back of the skull where they are interleaved with a series of very narrow and elongate feathers (61 mm), closely approaching the length of the skull (63 mm) and approximately twice the length of the adjacent neck feathers (34 mm). Dark tips are not observed on these elongate feathers, which also have a bristle-like aspect. However, with magnification, barbs are discernable closely appressed to the rachises near the tips of several of these feathers (Fig. 1C; Fig. S1 top left panel). Proximally, the barbs are very evenly spaced throughout their preserved lengths (Fig. S1 top left panel) implying that they may have been closed pennaceous with functional hooklets.

Ventral to the skull and just rostral to the orbit, a small patch of feathers darkens distally. These distal margins appear truncate or squared (Fig. 1D). Individual barb tips protrude slightly distal to these truncate edges similar to the preservation in banded feathers (Fig. 1D; Vinther et al., 2008), but unlike the recurved distal margin typical of many contour feathers or the dorsal crest. These characteristics in the gular feathers are consistent with the possible presence of differently colored, or white, feather tips that at least slightly surpass these darkened regions (Fig. 1). Better exposed on Slab A, at least two lines of these feathers extend slightly further down the neck, but missing sections make it impossible to determine how far they may have continued; one feather, similar in preserved patterning, is also visible closer to the omal region.

As previously reported, the primaries are extremely elongate (∼195 mm) and asymmetrically veined (Figs. 1A and 1D; Chiappe et al., 1999). An alula is not visible. The proximal primary rachis is broad and tapers gradually distally (e.g., 1.2 mm in diameter on the penultimate primary). Best exposed in this primary (of the right wing in Slab A), the rachis has dark edges and a lighter core (Figs. 1A and 1D). The remains of nine elongate primaries are visibly aligned in the right wing of the new specimen (∼195 mm). Each primary is associated with a major covert (primary covert lengths, distal to proximal: 48, 54, 66.5, 55, 51 mm). The coverts are generally more elongate near the wing tip and shorten toward the wrist. The leading edge primary is short (∼98 mm) (Fig. 1A). Two feathers that pass under the secondaries and are splayed to the side are interpreted as two additional primaries; they are aligned with the wrist rather than with the ulna (Fig. 1A). However, the potential that they represent secondaries cannot be ruled out. In one of the best specimens referred to *Confuciusornis* to preserve fine feather details, IVPP V 13156, 11 primaries are also present. A second line of minor primary coverts visible in Slab B is approximately two-thirds of the length of the major coverts (i.e., 37 mm on feather 4; Fig. 1A).

Secondaries and ventral secondary covert feathers of the right wing angle from the ulna toward the body as preserved (Fig. 1A). Approximately four elongate and closely-spaced feathers from this series are roughly aligned with the primaries. One feather, the most proximal preserved secondary, is further shifted toward the body but better exposed (length in Slab A: 101 mm; Slab B: 103 mm), and it may be missing a small part of its distal tip. The set of shorter, aligned feathers are interpreted as the major secondary coverts, and at least four sets of minor secondary coverts with dark tips of varying lengths are preserved (approximate lengths 64, 48, 39, 25 mm; Fig. 1A). In their loose organization, they resemble the secondary coverts of *Anchiornis* (Li et al., 2010). Spotted (perhaps dorsal) secondary covert feathers organized into distinct layers are known in only one other specimen (STM13-30) referred to the Confuciusornithidae, which is figured in Zheng (2009).

Short feathers extend the length of the tibia (Fig. 1A). More abbreviate feathers close to the proximal tarsometatarsus are also distinguishable in both the right and left foot. A small cluster of short, narrow feathers with dark tips is well exposed and preserved in Slab B close to the right tibia (Fig. S1 lower right). These feathers appear to be associated with the base of the tail; on the left side of the specimen, several small feathers with dark tips appear to be present but are poorly preserved (Fig. S1 upper right). Long, streamer-like rectrices are not preserved. However, there is breakage just distal to the tip of the pygostyle and the absence of streamers in life cannot be ascertained with confidence (Fig. S1 lower right).

### Analyses of microbodies

**Figure 3:**
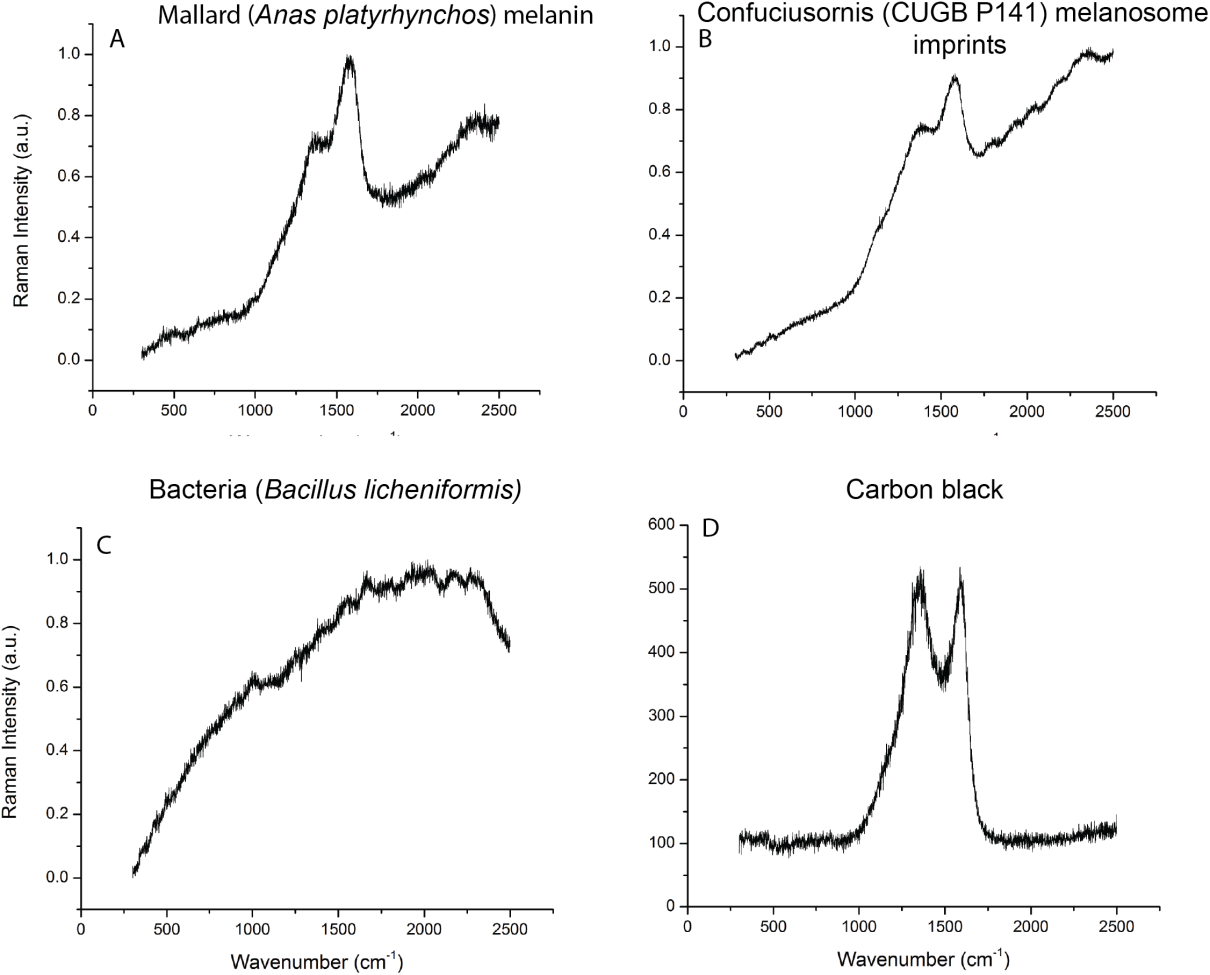
Raman spectroscopy data compared to melanin, keratin, and bacterial signatures. (A) Extant melanin from Mallard *Anas platyrhynchos*, (B) *Confuciusornis* specimen CUGB P1401 feathers, (C) the feather-degrading bacterium *Bacillus licheniformis*, and (D) carbon black.

Only one sample contained microbodies preserved in three dimensions, and the remainder contained microbody impressions (Fig. 2 and Fig. S4). We identified Raman signatures similar to eumelanin in modern bird feathers and a *Sepia* melanin standard in 24 of the 31 fossil samples (Fig. 2). The presence of these spectra even in samples with preservation of only melanosome imprints may be attributable to the residual presence of melanin in the matrix (see below). Why these signatures are present in some imprint samples but not others (see Fig. 2) is unclear and requires further investigation. The Raman results support melanin identity, but are not conclusive due to the similarity of these spectra to carbon spectra from fossil plants preserved as carbonaceous impressions (Peteya et al., 2017). Because it is unlikely that these plants contained melanin in life, we also tested the chemistry of our fossil samples using ToF-SIMS, which is commonly used to detect melanin in the fossil record (Colleary et al., 2015; Lindgren et al., 2014, 2015a, 2015b), and MALDI, which has not previously been employed for detecting fossil melanin (see Supplement). ToF-SIMS spectra from the *Confuciusornis* samples resembled spectra from the matrix (Fig. 3; Supplementary data). MALDI results were similarly inconclusive, as the fossil spectra lacked a melanin dimer peak present in extant avian and *Sepia* melanin samples at approximately 659.3 m/z (Fig. 5). Fossil MALDI results do not resemble spectra from carbon controls (Fig. S11).

**Figure 4:**
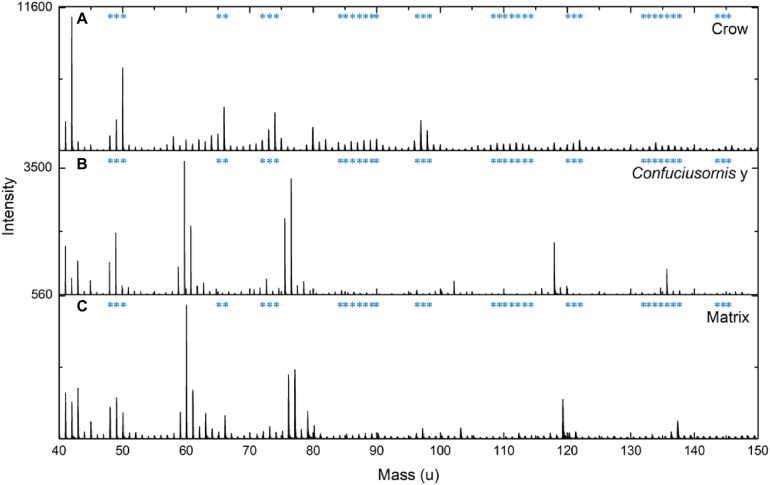
Comparison of ToF-SIMS spectra from extant and fossil melanin and a matrix control. ToF-SIMS results for fossil sample CUGB P1401 sample Y (A) compared to spectra from extracted crow melanin (B) and a CUGB P1401 matrix (C) sample. Asterisks indicate theoretical peaks for eumelanin from Lindgren et al. (2015b).

**Figure 5:**
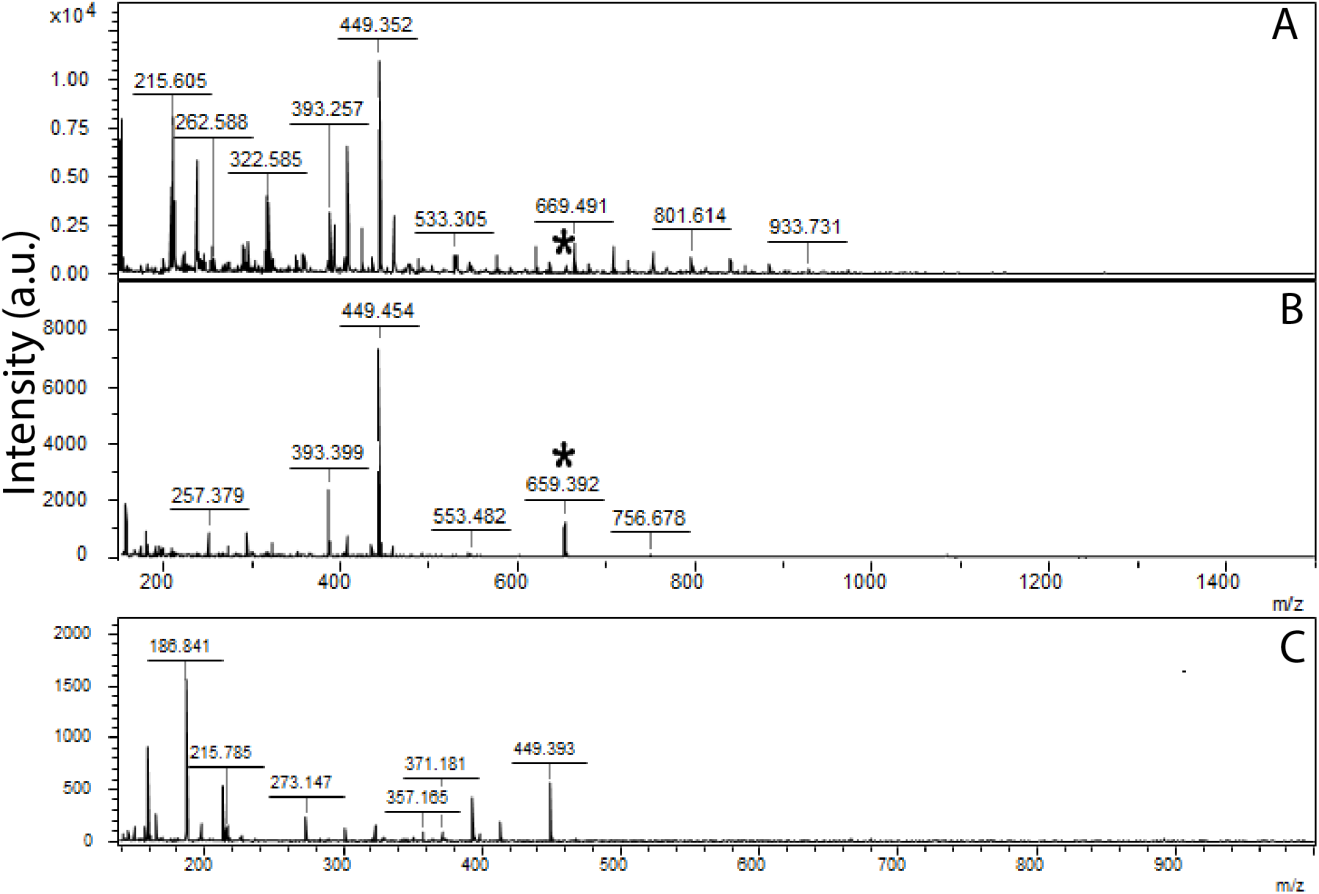
MALDI results form fossil and extant melanin. Compared to MALDI spectra from extracted eumelanin from Red-winged Blackbird feathers (A), a Sepia ink standard (B) and MALDI results for CUGB P1401 sample B1 (C). The asterisk indicates a peak for a melanin dimer plus a sodium ion, which is present in the modern melanin samples, but not CUGB P1401 samples.

A previous study of the confuciusornithid *Eoconfuciusornis* detected putative keratin and melanin in fossil feather samples using ChemiSTEM elemental analysis, suggesting that the identification of a keratinous matrix is pertinent to differentiating between melanosomes and bacteria in the fossil record (Pan et al., 2016). Raman spectroscopy did not detect keratin in the *Confuciusornis* samples (Fig. S5). However, Raman active molecules, which have stronger Raman signals due to greater numbers of carbon–carbon bonds (e.g., eumelanin), often overshadow other chemical signals. We did not test for keratin preservation using ToF-SIMS or MALDI. However, our results are generally consistent with eumelanin and inconsistent with bacterial preservation. Raman spectra from the fossil samples are dissimilar to spectra from the common extant feather bacterium *Bacillus licheniformis* (Fig. 3; Burtt & Ichida, 1999; Peteya et al., 2017). ToF-SIMS spectra from the *Confuciusornis* samples also do not resemble those reported for pyomelanin—a common modern bacterial melanin (Lindgren et al., 2015b), although minute quantities may not be detected. This chemical evidence coupled with the lack of mitotic fissures in any of the preserved microbodies, their restriction to the fossilized integument, and a lack of other evidence of a biofilm (e.g., mycelia; Borkow & Babcock, 2003) suggest that the preserved microbodies are unlikely to be fossil bacteria.

Preserved microbodies were homogenous in shape within 18 out of 32 samples (Fig. 1; Fig. S3; Table S2). However, eight samples, mostly from the crest, face, and spotted areas of the secondaries, contained microbodies with a high diversity of shapes, ranging from near-spherical to elongate and rod-like (Fig. 1; Fig. S4; Table S2). This single-sample diversity is not typically seen in extant feathers of a single color, where melanosomes within either barbs or barbules are not so heterogeneous (Durrer, 1977; Li et al., 2012). In these regions, barbs, and barbules may have contained different melanosome types, or color may have been patchy within or between feathers. Systematic variation of melanosome shape between barbs and barbules of extant birds has not been assessed. In *Anchiornis huxleyi*, these regions were interpreted as showing closely-spaced reddish brown and gray feathers (Li et al., 2010). Several samples contained sheets of round bodies that were significantly outside the range of melanosomes found in modern birds and varied nearly continuously in size (Fig. S6). The absence of mitotic fissures in these structures makes them inconsistent with bacterial identity. Nevertheless, in this specimen we refrain from assigning discrete colors to samples using the methods deployed in previous work (Li et al., 2010; Li et al., 2012). We speculate that the melanin-based plumage color of the new confuciusornithid was likely gray or black given the distribution and shape of preserved putative melanosomes; most body feathers show melanosomes that are uniformly long or oblong (Fig. 2). These results are consistent with previous reports of melanosome morphologies preserved in the body feathers of *C. sanctus* (Wogelius et al., 2011).

## Discussion

**Figure 6:**
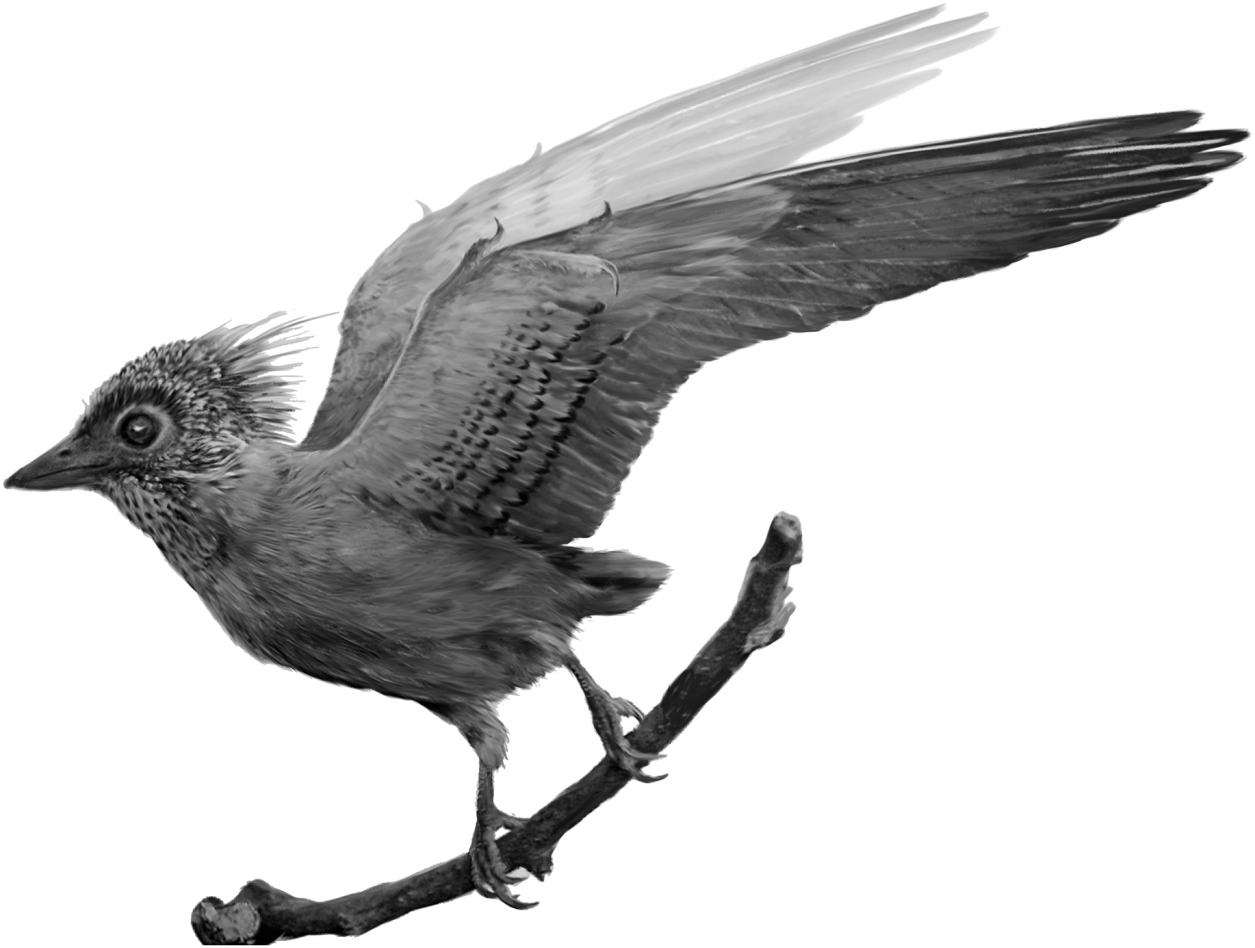
Reconstruction of the plumage in CUGB P140. Morphological data and probable eumelanin signatures are consistent darker preserved regions as associated with dark colors in the fossil (sampling regime explained in Fig. 1).

Of the hundreds of specimens referred to the Confuciusornithidae, only one specimen referred to *Confuciusornis* shows plumage patterning of the secondary covert feathers similar to the new specimen (Zheng, 2009). Tiny spots were illustrated on these coverts. The wing appears to be in dorsal view. Here, we find evidence for larger spots on these coverts and a distinctive head crest, patterned gular region, as well as possibly patterned tibial feathers (Figs. 1 and 6; Fig. S1). Original dark-light color patterning is clearly preserved in fine detail in this specimen (Fig. 1). Dark spots that range in length from 25 to 65 mm are preserved on the secondary covert feathers of the right wing on the part slab. Similar patterns preserved on the secondary covert feathers of *Anchiornis* and *Eoconfuciusornis* have been interpreted as original color patterning (Li et al., 2010; Zheng et al., 2017). Dark, crescent-shaped patterns are preserved along the crown of CUGB P1401, and distally box-like feathers are preserved along the gular region. We interpret these aberrant morphologies as differentially melanized color patterns rather than novel feather morphologies. The crown feathers likely had melanized distal edges while the remainder of the feathers, which consistently do not appear to be preserved, were unmelanized in life. Similarly, the distal ends of the gular feathers were unmelanized while the rest of the feathers were melanized. The unmelanized regions may have been white or otherwise pigmented by carotenoids or other pigments, although these were not chemically detected.

These patterns suggest that complexity of plumage patterning of Cretaceous birds may have exceeded the spangles of *Anchiornis* (Li et al., 2010) or tail stripes of *Sinosauropteryx* (Zhang et al., 2010) and indeed rivaled that of extant Aves, with potentially similar functions. The size and placement of the spots in northern flickers (Piciformes), barn owls (Strigiformes), and hoopoes (Bucerotiformes) approach those observed in the new confuciusornithid. In barn owls, males prefer females with larger spots (Dreiss & Roulin, 2010), indicating a potential role for sexual selection even in taxa in which dichromatism is not pronounced. Avian crests are used in numerous signaling contexts, from agonistic interactions (Brown, 1963) to courtship (Graves, 1990). Together, the presence of these features in this fossil specimen may indicate that plumage functioned as signals even early in avian evolution (Li et al., 2012). However, densely packed spots are also characteristic of camouflaged plumage (e.g., in caprimulgiform birds; Diamond & Bond, 2013). These patterns enable crypsis through matching of their plumage with the background (Stevens et al., 2009). In the new specimen, the admixture of differently-sized melanosomes, even in individual SEM images, and the overall diversity of melanosome shapes in the same samples may suggest the presence of numerous color patches in close proximity. For these reasons, a role for the spotted coverts in crypsis cannot be ruled out.

The lack of a significant chemical signal for melanin and the enigmatic melanosome preservation suggests that the original colors may be lost in this specimen. Raman spectra consistent with eumelanin were detected in approximately 77% of the fossil samples that also had preserved microbodies, but other chemical methods were inconclusive. It could be argued that the Raman spectrometer is detecting the carbonized remains of the *Confuciusornis* feathers, given the lack of a conclusive melanin signature using ToF-SIMS and MALDI, the similarity between the Raman signature for eumelanin and that of carbonaceous plant fossils (Peteya et al., 2017), and the differential Raman signal between fossil samples with similar microbody preservation (Fig. S4). However, the MALDI results for the fossil samples did not resemble the MALDI results for any of the carbon controls (Figs. S10 and S11). If the Raman signal is indeed a carbon signal, then the carbon source was either keratin, a pigment, or possibly decay microbes. We have ruled out preservation of microbes. Sections of the gular and crown feathers were not preserved, suggesting that these feathers were unmelanized in life (Vinther, 2015). If the source of the carbon was keratin, we would expect these sections to be carbonized as well as the originally melanized regions, but this was not true. Given melanosome preservation in darkly-colored feathers, the original source of a carbon signal would thus likely have been melanin. The lack of eumelanin signals for ToF-SIMS or MALDI may be due to diagenetic alteration of the original melanin (Colleary et al., 2015). Alternatively, the chemical signal for melanin could be too weak for the other chemical techniques to detect. Indeed, melanosomes in all of the analyzed samples were found as imprints, which likely preserve chemistry with less fidelity than three-dimensionally preserved melanosomes. Melanin traces have been detected in another *Confuciusornis* specimen using trace metal-based chemical techniques (Wogelius et al., 2011) and minute amounts of eumelanin have been detected in a Carboniferous fish fossil using alkaline hydrogen peroxide oxidation (Tanaka et al., 2014).

Although the majority of the microbodies preserved in the fossil samples fall within the morphological range of extant melanosomes, some samples contained microbodies outside of this range. Our current knowledge of melanosome taphonomy suggests that melanosome may change in size, but not shape, in feathers under experimental taphonomic conditions (McNamara et al., 2013; Colleary et al., 2015). The aberrant microbodies preserved in our *Confuciusornis* samples may represent novel melanosome morphologies or preservational artifacts that should be investigated further. Taphonomic mode thus merits further inquiry.

## Conclusions

The elaborate spotting on this specimen exceeds that found in exceptionally-preserved troodontids and compsognathids and rivals that in modern birds, suggesting that plumage patterns evolved greater complexity through avian evolution. This hypothesis remains to be tested as more exceptionally-preserved specimens are described.

## Supplemental Information

10.7717/peerj.5831/supp-1 Supplemental Information 1 Supplementary Materials and Methods.

10.7717/peerj.5831/supp-2 Supplemental Information 2 Raw Raman spectroscopy readings of fossil and extant melanins. Each column is a Raman spectroscopy reading from an extant or fossil melanin sample.

10.7717/peerj.5831/supp-3 Supplemental Information 3 Raw ToF-Sims readings from extant and fossil melanins. Each column is a Tof-SIMS reading from an extant or fossil melanin sample.

10.7717/peerj.5831/supp-4 Supplemental Information 4 Raw Maldi-ToF readings from extant and fossil melanins. Each column is a Maldi-ToF reading from an extant or fossil melanin sample.

10.7717/peerj.5831/supp-5 Supplemental Information 5 Tof-Sims data summary table. Summary of peak locations and values in the Tof-Sims data.

## Additional Information and Declarations

### Data Availability

The following information was supplied regarding data availability: The raw data are provided in the Supplemental Files.
