# Supplementary material for: Elaborate plumage patterning in a Cretaceous bird
Source: PeerJ. 2018 Nov 2;6:e5831. doi: 10.7717/peerj.5831 (PMC6216952; doi:10.7717/peerj.5831)
Supplement: Supplemental Information 1 [file peerj-06-5831-s001.pdf]

SUPPLEMENTARY MATERIALS AND METHODS

Taxonomic status of CUGB 1401

CUGB P1401 (China University of Geosciences, Beijing) is differentiated from *Eoconfuciusornis zhengi* by possessing a much larger deltopectoral crest with a foramen at its centre and a posterior sternal margin with diminutive trabeculae and twin concavities (incisurae; Fig. S1) rather than with a straight to convex margin (Zhang et al., 2008). A large deltopectoral crest (Fig. S1) is seen in confuciusornithid specimens referred to *Confuciusornis*, *Changchengornis* and *Jinzhouornis* (Chiappe et al., 1999; Zhang et al., 2008).

The new specimen is differentiated from *Changchengornis hengdaoziensis* by the presence of a large round foramen perforating this large deltopectoral crest (Fig. S1; Ji et al., 1999). It shares this perforated crest with all named species of *Confuciusornis* and *Jinzhouornis.* The two named species of *Jinzhouornis* (Hou, 2002) have alternatively been proposed to be junior synonyms of *Confuciusornis sanctus* (Chiappe et al., 2007, 2008; Marugán-Lobón et al., 2011) or diagnosably distinct (Li et al., 2010a). One concern with the taxonomic status of *Jinzhouornis* is that all characters proposed to be diagnostic for this genus are also present in *Confuciusornis* with the exception of an elongate rostrum. The two elements of *Jinzhouornis yixianensis* that could be reliably measured were nearly identical in proportions to other *Confuciusornis* specimens. Furthermore, there are abundant specimens of *Confuciusornis sanctus* with rostri of the proportions of *Jinzhouornis zhangjiyinensis* and *Jinzhouornis yixianensis* (e.g., Chiappe et al., 1999, figs. 8, 15; GMV 2130), which may support previous recommendations of junior synonym status. Furthermore, the skull of the holotype of *Jinzhousornis zhangjiyingensis* does not appear confidently associated with the rest of the skeleton as the cervical series is conspicuously truncated by a large crack separating it from the skull.

The new specimen is differentiated from *Confuciusornis feduccai* and *Confuciusornis dui* but similar to *Confuciusornis sanctus* in the presence of relatively deep rather than shallow incisures on the posterior sternal margin; it also differs from these taxa in size (Fig. S1; Table S1; Zhang et al., 2009). The holotype of *C. chuongzhous* consists of only a partial hind limb. It and that of *C. suniae* are identical in relative proportions and morphologies to *C. sanctus* (Table S1; Chiappe et al., 1999, 2008; Marugán-Lobón et al. 2011). *Confuciusornis jianchangensis* is slightly smaller than CUGB P1401 based on published measurements (Li et al., 2010*a*). While its taxonomic status has not been revisited, it suffers the same issues identified for previously proposed species of the Confuciusornithidae (Chiappe et al., 2008; Marugán-Lobón et al., 2011).

Table S1. Measurements of the new specimen (CUGB 1401) compared to holotype and referred confuciusornithid specimens. Direct measurements for 8 specimens were added to the supplementary data table from Chiappe et al. (2008). Data on the holotype specimens of *Confuciusornis feducciai* and *Confuciusornis dui* are from Zhang et al. (2009), and those for *Changchengornis hengdaoziensis*, from Ji et al. (1999). Values are sorted by femur length, followed by humerus and ulna length.

Table S1.							
**Specimen**	Taxon referred (Chiappe, 2008) or h	**fem.**	**hum.**	**uln.**	**rad.**	**tib.**	**rectric.**
GMV-1	*Confuciusornis sanctus*	–	–	–	34	–	Present
GMV-78	*Confuciusornis sanctus*	–	–	44.63	–	–	Indet.
GMV-21-1	*Confuciusornis sanctus*	–	64.6	52.4	51.9	63.3	Present
GMV-15	*Confuciusornis sanctus*	–	61.8	54.5	–	64.3	Present
GMV-32	*Confuciusornis sanctus*	–	54.95	–	43.8	56.4	Present
D1196	*Confuciusornis sanctus*	–	53.16	46.5	41.36	52.54	Present
GMV-52	*Confuciusornis sanctus*	–	52.7	45.4	43.7	53.7	Present
GMV-67-2	*Confuciusornis sanctus*	–	52.4	–	42.9	49.5	Absent
GMV-16	*Confuciusornis sanctus*	–	48.55	40	38.7	–	Present
SMNK-Pal.6413	*Confuciusornis sanctus*	–	48.31	42.16	40.32	51.57	Indet.
GMV-16	*Confuciusornis sanctus*	–	47.08	–	37.17	–	Indet.
GMV-2146	*Confuciusornis sanctus*	–	42.45	35.62	34.68	–	Absent
GMV-2031	*Confuciusornis sanctus*	–	41.44	34.96	35.05	–	Absent
BSP 1999 I 15	*Confuciusornis sanctus*	59.47	68.55	58.04	57.8	70.24	Present
D2454	***Confuciusornis feducciai***	59	78.5	75		69	Present
IVPP 11370	*Confuciusornis sanctus*	58.89	68.14	58.21	54.89	69.12	Present
D2454	*Confuciusornis sanctus*	58.49	78.09	69.96	–	68.87	Absent
IVPP V13175 - pre	*Confuciusornis sanctus*	58.44R/58.7	~61.13R/~6	58.57R/56.0	54.36R/52.1	66.96R/66.4	Present
GMV-2132	*Confuciusornis sanctus*	57.63	68.37	56.95	55.7	65.22	Indet.
GMV-55-1	*Confuciusornis sanctus*	57.5	69.25	59.15	57.25	66.6	Present
MCFO-0589B	*Confuciusornis sanctus*	56.28	–	59.14	–	66.64	Present
GMV-26	*Confuciusornis sanctus*	56.1	65.7	54.8	52.3	66.4	Present
SMF AV 420	*Confuciusornis sanctus*	55.97	–	–	–	–	Indet.
GMV-7	*Confuciusornis sanctus*	55.91	–	–	–	–	Indet.
GMV-54	*Confuciusornis sanctus*	55.9	67.7	57.45	53.6	65.75	Present
GMV-59-1	*Confuciusornis sanctus*	55.7	65.75	57	53.1	67.25	Present
GMV-53	*Confuciusornis sanctus*	55.6	67.65	60.6	55.85	65.6	Present
IVPP V13175 -pres	*Confuciusornis sanctus*	55.46R/53.6	67.8R/65.62	59.22R/57.4	55.86R/54.5	67.85R/67.3	Present
LACM 153346	*Confuciusornis sanctus*	55.43	59.51	51.65	50.75	63.56	Indet.
JME/UKr 1996/15	*Confuciusornis sanctus*	55.4	–	55.81	53.55	62.4	Indet.
GMV-46	*Confuciusornis sanctus*	55.4	66.9	56.1	52.55	67	Absent
NHMW1997z/0000	*Confuciusornis sanctus*	55.23	66.4	57.37	56.81	66.67	Absent
LPM0228C	*Confuciusornis sanctus*	55.19	61.15	55.6	50.96	63.96	Absent
IVPP V13178	*Confuciusornis sanctus*	55.1R/54.2L	66.0R/66.6L	59.4R/59.1L	55.5R	64.8R/64.4L	Present
IVPP 11640	*Confuciusornis sanctus*	55	64.94	54.86	50.81	65.42	Absent
GMV-59-2	*Confuciusornis sanctus*	55	63.75	54.45	49.8	61.1	Present
GMV-20-2	*Confuciusornis sanctus*	54.8	69.5	58.6	52.1	66.35	Present
GMV -43	*Confuciusornis sanctus*	54.6	67.2	–	54.6	66.5	Present
SMF AB 416	*Confuciusornis sanctus*	54.53	65.29	–	54.58	63.64	Present
GMV-50	*Confuciusornis sanctus*	54.45	62.9	56.2	52.75	63.8	Present
GMV-67-1	*Confuciusornis sanctus*	54.05	66.3	–	57.2	66.15	Present
GMV-39	*Confuciusornis sanctus*	53.95	63.7	–	–	64.8	Present
GMV-62	*Confuciusornis sanctus*	53.7	61.6	51.35	–	64.1	Present
MCFO-0589A	*Confuciusornis sanctus*	53.21	61.66	56.27	52.37	64.34	Present
GMV-41	*Confuciusornis sanctus*	53.16	62.57	54.38	52.08	61.6	Indet.
IVPP13156	*Confuciusornis sanctus*	53.16	60.97	53.52	–	60.81	Absent
GMV-25	*Confuciusornis sanctus*	53	60.45	53.5	50.7	58.1	Present
GMV-66-2	*Confuciusornis sanctus*	52.95	65.45	55.4	51.8	63.7	Present
GMV-33	*Confuciusornis sanctus*	52.9	58.5	52.1	50.2	63.1	Present
TMP 981401	*Confuciusornis sanctus*	52.43	62.77	53.93	51.99	63.33	Present
GMV-2154	*Confuciusornis sanctus*	52.35	63.65	53.4	52.05	63	Present
GMV-69-1	*Confuciusornis sanctus*	51.9	64	55.4	51.7	61.5	Present
GMV-69-2	*Confuciusornis sanctus*	51.8	64.5	54.2	51.8	61.5	Present
LPM0228B	*Confuciusornis sanctus*	51.71	60.89	49.18	–	–	Absent
GMV-19	*Confuciusornis sanctus*	51.5	62	52	48.5	59.3	Absent
D2151	*Confuciusornis sanctus*	50.94	66.9	53.83	50.39	63.16	Absent
LPM0228A	*Confuciusornis sanctus*	50.87	–	54.39	–	63.98	Absent

Table S1 Continued.

GMV-73	*Confuciusornis sanctus*	48.4	58.85	50.7	48.75	57.85	Present
GMV-36	*Confuciusornis sanctus*	48	55	47.35	44.2	53.7	Present
GMV-77	*Confuciusornis sanctus*	47.9	56.15	—	45.65	54.85	Present
GMV-61	*Confuciusornis sanctus*	47.6	—	—	—	60.7	Present
LPM0229 (lt)**	*Confuciusornis sanctus*	47.12	57.23	49.42	46.86	58.2	Present
GMV-2133	*Confuciusornis sanctus*	46.85	53.94	47.66	48.18	54.04	Present
IVPP 11308	***Confuciusornis suniae***	46.79	53.18	46.46	45.96	55.83	Indet.
SMF AV 417	*Confuciusornis sanctus*	46.73	50.73	43.85	40.49	53.68	Indet.
TMP 981402	*Confuciusornis sanctus*	46.7	51.33	—	—	52.67	Present
JME/UKr 1997/1	*Confuciusornis sanctus*	46.1	51.79	46.6	43.9	53.29	Present
IVPP 11374	*Confuciusornis sanctus*	45.86	51.46	45.84	44.4	53.33	Absent
IVPP 11375	*Confuciusornis sanctus*	45.74	53.28	44.68	42.87	52.76	Present
GMV-8	*Confuciusornis sanctus*	45.7	53.25	45.3	43.9	52.1	Present
IVPP 100921	*Confuciusornis sanctus*	45.58	55.76	49.58	48.72	53.46	Absent
GMV-40	*Confuciusornis sanctus*	45.45	56.3	47.2	44.2	54.1	Present
IVPP V13168	*Confuciusornis sanctus*	45.31	51.73	43.27	43.84	52.72; 53.2	Present
GMV-2153-1	*Confuciusornis sanctus*	45.25	52.9	44.2	41.55	54.5	Present
GMV-2155	*Confuciusornis sanctus*	45.15	51.75	—	43	52.85	Present
LPM0012	*Confuciusornis sanctus*	45.03	50	41.2	42.94	53.24	Present
GMV-2149	*Confuciusornis sanctus*	45	53.5	—	—	—	Indet.
GMV-2150	*Confuciusornis sanctus*	45	51.15	44.05	39.9	51.2	Absent
LL.12418	*Confuciusornis sanctus*	44.93	50.06	42.93	41.1	49.76	Indet.
GMV-44	*Confuciusornis sanctus*	44.9	52.1	44.8	42.45	55	Present
GMV-6	*Confuciusornis sanctus*	44.74	52.86	—	43.17	—	Indet.
IVPP 11372	*Confuciusornis sanctus*	44.73	52.77	47.07	44.62	51.09	Absent
GMV-56-2	*Confuciusornis sanctus*	44.5	51.1	45.25	43.2	53.55	Present
GMV-9	*Confuciusornis sanctus*	44.5	50.95	—	43.15	52.2	Present
JME/UKr 2005/1	*Confuciusornis sanctus*	44.08	59.31	46.68	44.4	54	Present
D2860	*Confuciusornis sanctus*	44.04	53.75	46.84	—	54.76	Present
GMV-14	*Confuciusornis sanctus*	44	50.2	46.2	44.2	52.2	Present
GMV-20-1	*Confuciusornis sanctus*	43.95	52.85	46.9	44.6	53.7	Present
D2859	*Confuciusornis sanctus*	43.93	51.8	44.83	42.23	50.82	Present
GMV-66-1	*Confuciusornis sanctus*	43.9	51.9	45.1	—	53.05	Present
**CUGB P1401**	**Confuciusornis sp.**	43.9	51.2	44.75	45.1	52.5	Absent
GMV-2151	*Confuciusornis sanctus*	43.9	50.9	—	—	—	Indet.
GMV-1	*Confuciusornis sanctus*	43.8	49.8	45.2	43.1	50.4	Present
IVPP10918	***Confuciusornis sanctus***	—	49.6	—	45.77	—	Present
SMF AB 412	*Confuciusornis sanctus*	43.73	—	42.81		50.4	Indet.
GMV-60	*Confuciusornis sanctus*	43.7	52.9	44.9	43.7	—	Present
CAGS 1	*Confuciusornis sanctus*	43.66	55.08	—	49.67	47.73	Present
BPV-2066	*Confuciusornis sanctus*	43.65	51.57	45.39	42.53	53.03	Indet.
NBM 258	*Confuciusornis sanctus*	43.44	51.75	44.05	41.15	52.09	Absent
LPM0229 (rt.)**	*Confuciusornis sanctus*	43.34	47.43	37.95	38.03	51.69	Present
GMV-56-1	*Confuciusornis sanctus*	42.8	50.1	45.5	41.3	51.95	Present
PMO.161.632	*Confuciusornis sanctus*	42.65	50.88	45.22	39.9	51.87	Absent
GMV-2	*Confuciusornis sanctus*	42.4	51.25	44.6	42.6	52.95	Present
IVPP 12352*	***Jinzhouornis zhangjiyinensis***	41.78	53.8	45.39	—	49.11	Present
GMV-2030	*Confuciusornis sanctus*	41.78	47.78	40.73	38.87	48.7	Indet.
MB.Av.1168-1171	*Confuciusornis sanctus*	41.71	45.51	41.72	—	48.88	Absent
IVPP V14412	**Jinzhouornis yixianornis**	-	46.6(L)	~46.4	-	~46.6	Indet.
MCFO -0374	*Confuciusornis sanctus*	41.4	47.54	41	38	46.85	Present
SMF AV 419	*Confuciusornis sanctus*	—	41.3	38.51	—	—	Absent
GMV-2153-2	*Confuciusornis sanctus*	37.7	44.7	39	36.7	—	Present
IVPP11977	Eoconfuciusornis	37.13L/35.4	~29.26	33.64	~31.42	41.83L/41.5	Present
IVPP 11553	***Confuciusornis dui***	35	42	39		41	Present
GMV-101	*Confuciusornis sanctus*	34.38	—	—	36.63	39.21	Indet.
GMV-30	*Confuciusornis sanctus*	33.4	40.85	35.8	—	40.9	Present
GMV-2129	***Changchengornis hengdaoziensis***	33.24	33.53	31.97	30.71	36.86	Present
LPM0233	*Confuciusornis sanctus*	32.44	41.01	—	—	36.85	Present

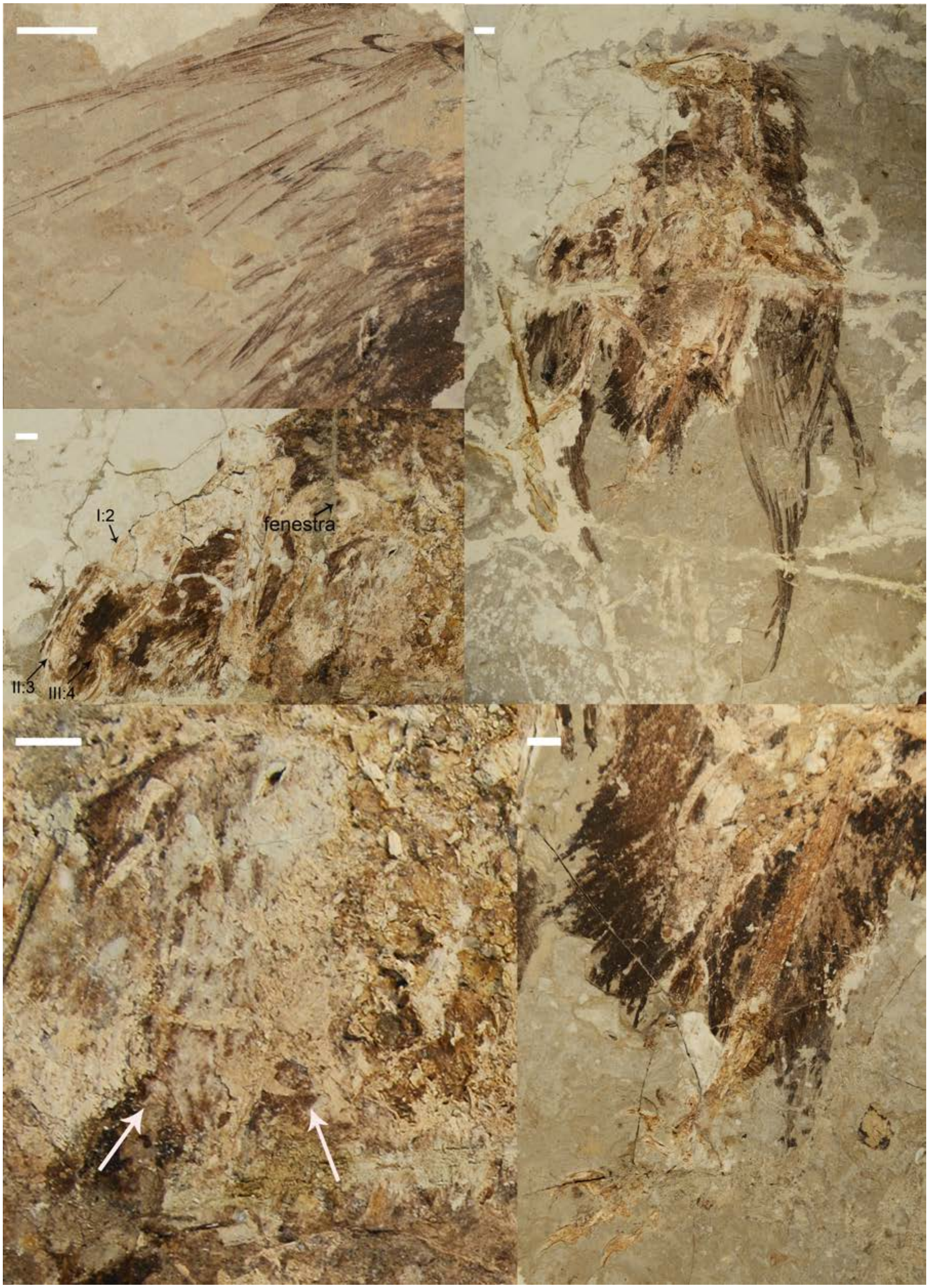

Fig. S1. Anatomical and feathering details of CUGB 1401. Clockwise from upper left: Closeup of the pinnate structure of the bristle feathers in the primary slab (Slab A); the counter slab, Slab B; close up of the short proximal tarsometatarsal feathers as well as tibial and tail feathering in Slab B; morphology of the posterior margin of the sternum

showing paired incisurae (arrows); pectoral girdle showing the enlarged ungual on manual digit I:I and the fenestra in the large deltopectoral pectoral crest of the humerus. Scale bars: Top right: 1cm, others 5mm.

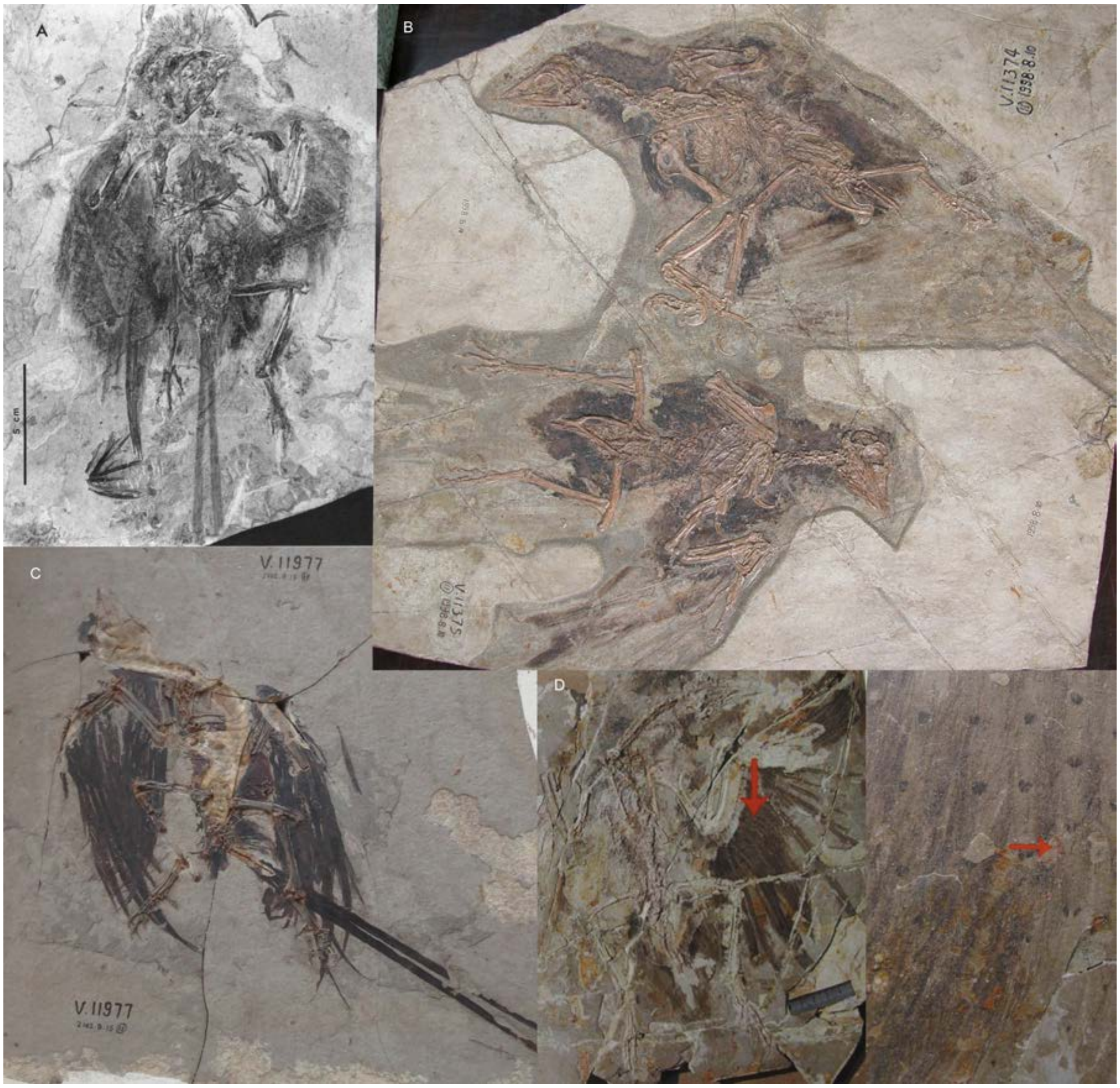

Fig. S2. Preserved feathering in specimens referred to *Confuciusornis sanctus* illustrating plumage differences from CUGB 1401 including the absence of an elongate patterned crest: A) *Chanchengornis* holotype specimen as illustrated in *2*) IVPP 11374, 11375 referred to *Confuciusornis sanctus*, C) *Eoconfuciusornis* holotype specimen *1*, D) the one other specimen referred to *Confuciusornis* showing secondary coverts with spangles as illustrated in Zheng (2009).

Melanosome sampling and assessment of morphology

We collected data on melanosome morphology from the fossil as previously described (Li et al., 2010b), with the exception that we took two (rather than one) samples from each location. Each of the samples was randomly assigned to either morphological (SEM) or chemical (Raman) analysis, such that we obtained both types of data from the same location on the fossil (see Fig. S3; Table S3 for details). The fossil was not exposed to glues or other chemicals prior to analysis.

Morphological samples were sputter-coated with silver and viewed on a ZEISS SUPRA-55 VP field emission SEM at China University of Geosciences, Beijing. Melanosomes were preserved in both three dimensions and as moldic impressions. For consistency, and to avoid potential differences between the two preservational styles (Clarke et al., 2010), we only measured impressions. We measured length and diameter of fossil melanosomes from these images using ImageJ and used these measurements to calculate morphological variables (mean, coefficient of variation, skewness of length, diameter and aspect ratio) as before. We visually examined images (Fig. S4) and plotted mean melanosome measurements against those of extant samples (Fig. S6; Li et al., 2012).

SUPPLEMENTARY RESULTS

Melanosome morphology

Six samples (o, r, w, a1, e1, k1) were outside the range of length, diameter or both for extant melanosome samples (Fig. S6). All of these samples were characterized by large, round morphologies and (in some cases) unusual honeycomb-like arrangements (e.g., sample k1, Fig. S4). In some cases (e.g., sample o, Fig. S4), these morphologies were co-localized with more rod-like morphologies that were of the typical size for melanosomes. Although both morphology and distribution in the remainder of the samples were in the range of modern melanosomes, these unusual features suggest that this fossil may have been preserved in a way that distorted melanosome features or introduced artifacts. We therefore did not use morphology to reconstruct colour of this specimen. These results support the need for caution in colour reconstruction (McNamara et al., 2013), but do not invalidate prior or future work using more conventionally preserved specimens.

Raman analyses

We compared Raman spectra from the fossil and matrix samples to extant melanin extracted from modern bird feathers, the common keratinolytic bacterium *Bacillus licheniformis*, carbon controls, including carbon black and fossil plants (Peteya et al., 2017), as well as bituminous coal and graphite as additional carbon controls and squid (*Sepia*) melanin as an additional melanin control. Raman spectra of all but one extant melanin sample were highly similar to one another and to previous spectra of eumelanin (Figs. 3, S3-4; Galván et al., 2013). They were characterized by a large peak at ~1578 cm/-1 and a smaller one at ~1360 cm/-1. A third, shorter peak was present in avian

melanin samples at ~1178 cm/-1 but at ~988 cm/-1 in *Sepia*. Spectra were characterized by numerous sharp peaks for keratin and by weak background peaks for the bacteria and matrix, and in all cases were dissimilar to those of extant melanin (Figs. 3, S3-4; Table S2-3). Peaks in the Raman spectra from the fossil samples closely matched those of extant eumelanin, but not those of keratin, bacteria or matrix (Figs. 3, S3-4; Table S2-3). Peaks for carbon black, bituminous coal, and graphite also differed, both in the location of the second main peak (~1590 cm/-1) and peak morphology (Figs. 3, S3-4; Table S3). However, fossil plants that likely lacked eumelanin have been shown to have similar Raman spectra to eumelanin, so Raman spectroscopy remains an inconclusive technique without the support of other chemical or morphological techniques (Peteya et al., 2017).

Raman curves from pheomelanic feathers of Rhode Island Red rooster *Gallus gallus* had numerous weak peaks, and none matched those of the other samples (Fig. S5). Previous studies showed distinctive Raman patterns for synthetic pheomelanin (Galván et al., 2013), but whether they are also characteristic of natural pheomelanin is only weakly supported. Indeed, the chemical distance of synthetic from natural eumelanin (Liu et al., 2014, Xiao et al. 2018) suggests that results from synthetic melanin should be interpreted with caution. Additionally, while Raman spectra from natural pheomelanin sources have been reported (Galván and Jorge, 2015; Galván et al., 2017), these signals are weak and difficult to characterize. We have tested pheomelanized samples extracted from Rhode Island Red rooster feathers and orange zebrafinch cheek feathers (in which pheomelanin composes over 99% of the total melanin (McGraw and Wakamatsu, 2004)) using both a green 532 nm laser (Figure S5) and an IR 785 nm laser, none of which gave distinctive pheomelanin peaks. In any case, our Raman spectral patterns were consistent across black, brown, and iridescent samples (Figure S5), including some that are known to contain a percentage of pheomelanin (Liu et al., 2014). The broad peaks of eumelanin may mask pheomelanin peaks in these samples, although the lack of distinctive pheomelanin peaks in feathers with greater concentrations of pheomelanin makes this unlikely. Moreover, the inability to detect eumelanin/pheomelanin mixtures using Raman spectroscopy suggests that other techniques (e.g. ToF-SIMS (Lindgren et al., 2014) or VUV-LDMS (Liu et al., 2014)) should be used in conjunction with Raman for distinguishing colours based on melanin chemistry. Indeed, the latter technique discriminates black from brown colours reasonably well. While chemistry from iridescent feathers is highly variable (Liu et al., 2014) their morphology is consistent (Li et al., 2012), suggesting that a combination of both morphological and chemical data may be a promising avenue in the further reconstruction of fossil colour.

Time-of-flight secondary ion mass spectrometry1

ToF-SIMS spectra showed peaks at the theoretical masses for eumelanin for all samples tested, including the extant samples, the CUGB P1404 fossil samples, and the matrix sample. Our PCA of the standardized relative intensities of all peaks did not separate the matrix sample from the fossil samples, although the wild turkey melanin sample also plotted with the CUGB P1404 samples (Figure S8). We were therefore unable to corroborate the presence of preserved eumelanin using PCA. Additionally, Colleary et al. (2015) showed that standardized intensities of fossil melanin ToF-SIMS spectra do not plot with modern eumelanin intensities and that experimentally matured samples bridge the gap between modern and fossil melanin samples. We could not include matured

samples in our analysis, so a gap between modern and fossil samples would be unsurprising if eumelanin is preserved. None of the ToF-SIMS spectra resemble previously reported pyomelanin – a microbial melanin – spectra (Lindgren et al., 2015a).

Matrix-assisted laser desorption/ionization mass spectrometry

Modern melanin samples, including the red-winged blackbird and *Sepia* melanin, presented a single peak at a mass of approximately 659.3 m/z (Fig. S9), which represents the combined masses of a melanin dimer and a sodium ion from the extraction buffer. This peak was also present in the fossil test sample, but not the carbon controls, the fossil plant samples, nor any of the CUGB P1401 samples (Figs. S10, S11). Some of the CUGB P1401 samples (b1, d1, e1, y) had a peak at a mass of approximately 357.1 m/z that could be a potassinated melanin monomer, but this peak was not present in either of the modern melanin samples. The 357 m/z peak is more likely an impurity. Additionally, MALDI spectra from the CUGB P1401 samples did not match any of the carbon controls. The chemical results for the *Confuciusornis* samples are therefore inconclusive. All other peaks present in the fossil samples and modern melanin samples can be attributed to salt cluster ions or other buffer-related contaminants.

Although it did not find melanin in the *Confuciusornis* samples, MALDI may be useful for future studies in fossil melanin, given that our test fossil sample did yield the 659.3 m/z eumelanin peak. However, this technique requires grinding the fossil samples, which are usually composed of a tiny bit of surface fossil material with underlying minerals, with a matrix, so larger fossil samples may be necessary to separate a melanin signal from the rock and matrix signals.1

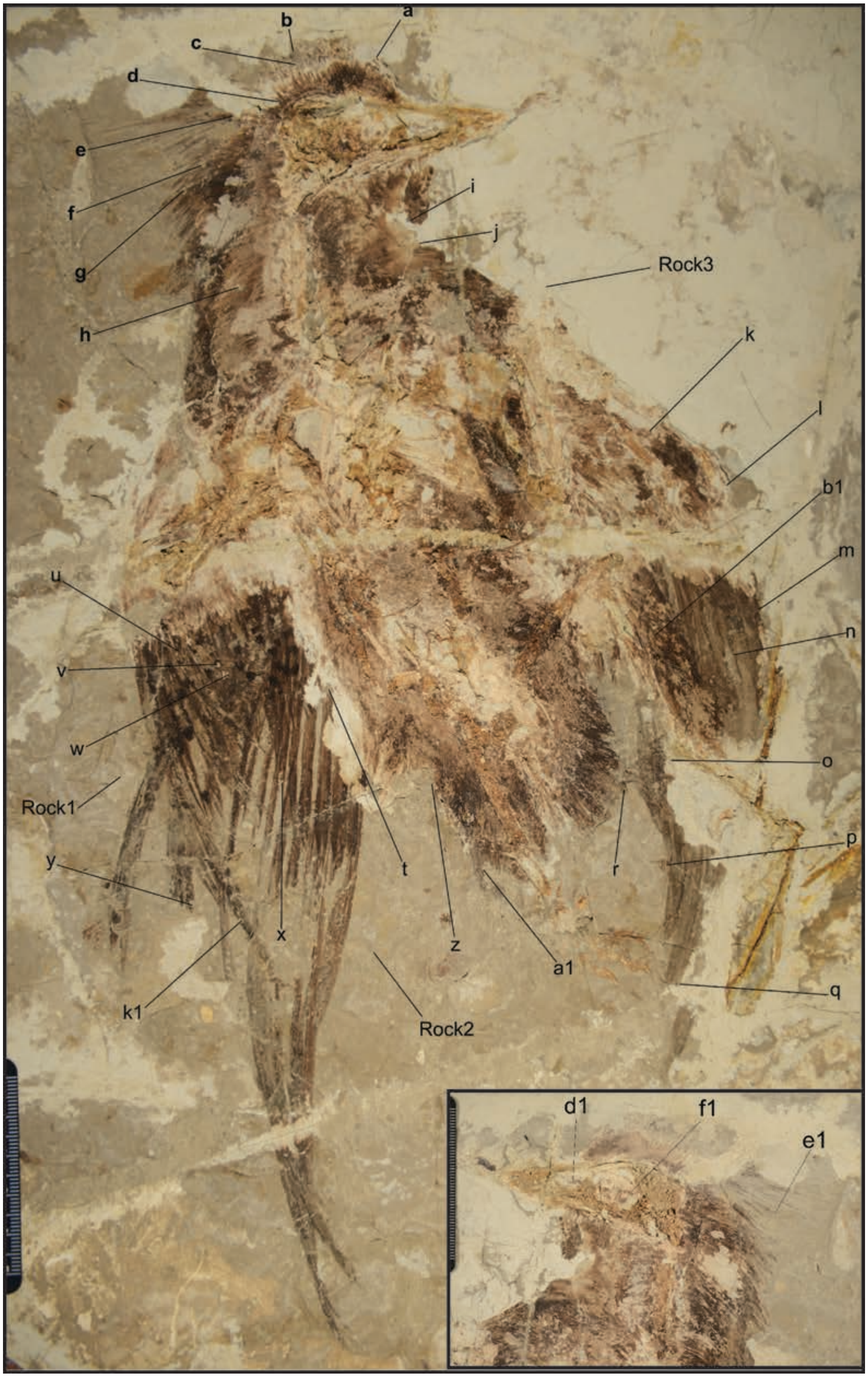

Fig. S3. Specimen CUGB 1401 with locations of samples marked. Sample codes are consistent throughout the manuscript. Included scales are in mm.

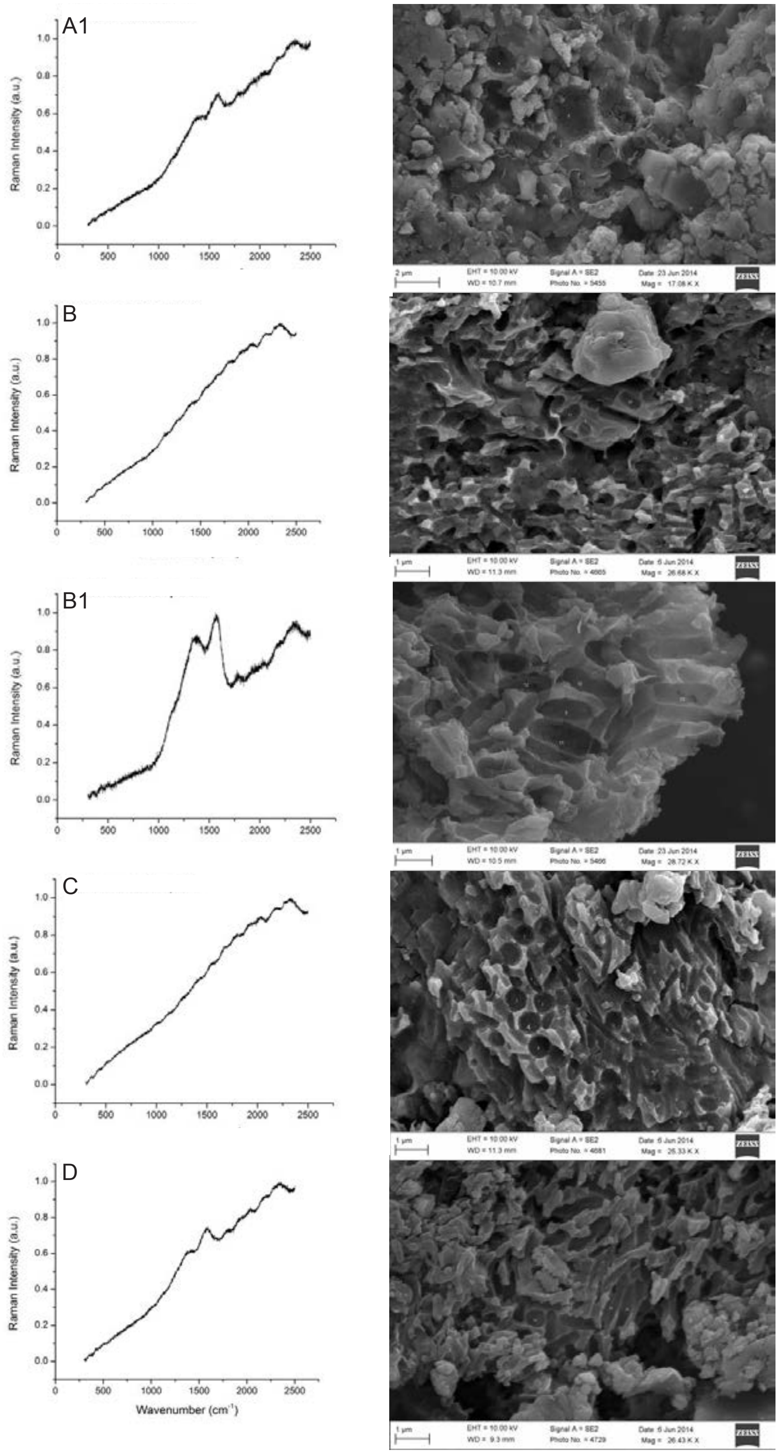
A1
B
B1
C
D
Raman Intensity (a.u.)
Wavenumber (cm-1)

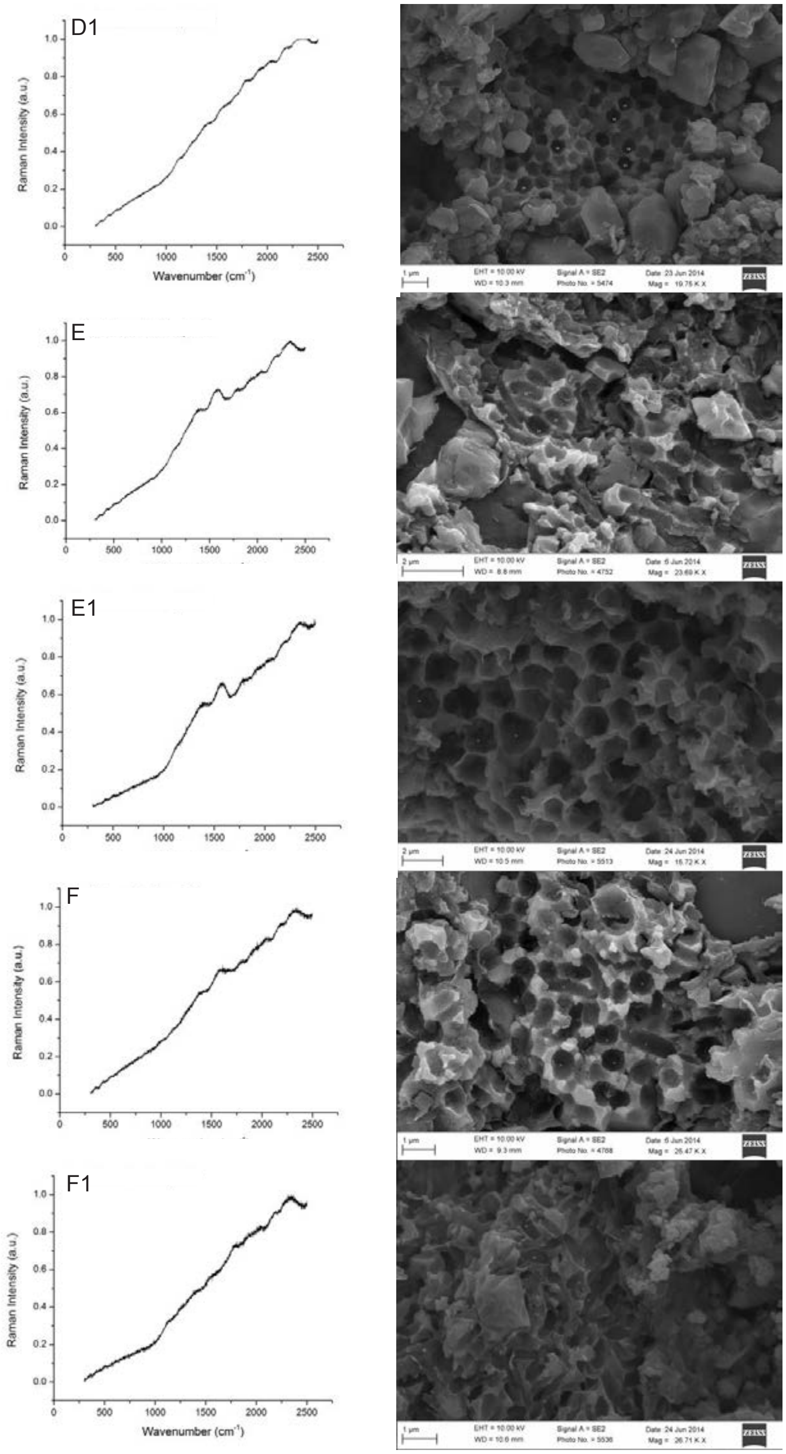

D1
E
E1
F
F1
Raman Intensity (a.u.)
Wavenumber (cm^{-1})

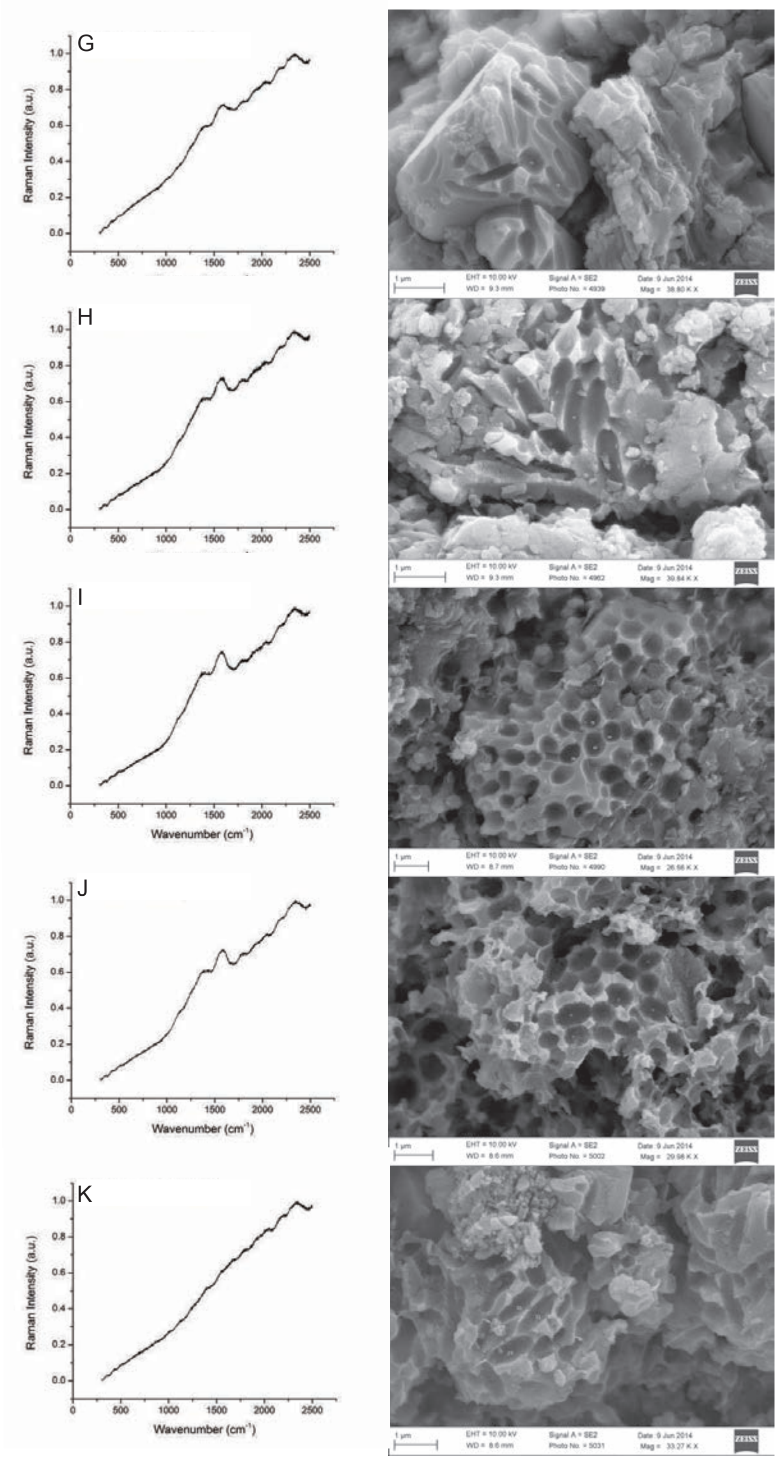
G
H
I
J
K
Raman Intensity (a.u.)
Wavenumber (cm^{-1})
0.0
0.2
0.4
0.6
0.8
1.0
0
500
1000
1500
2000
2500
1 μm
EHT = 10.00 kV
Signal A = SE2
Date: 9 Jun 2014

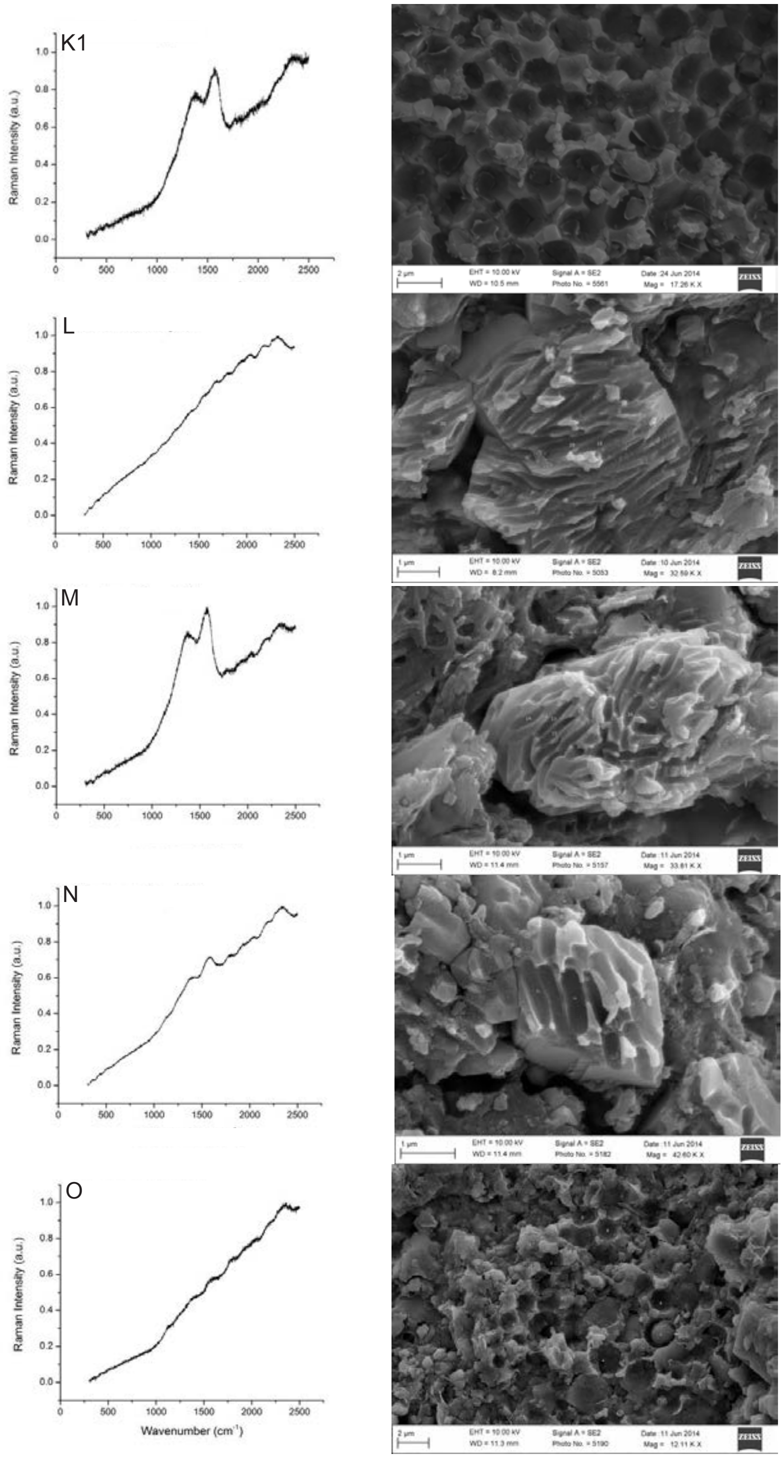
K1
L
M
N
O
Raman Intensity (a.u.)
0.0
0.2
0.4
0.6
0.8
1.0
0
500
1000
1500
2000
2500
Wavenumber (cm-1)

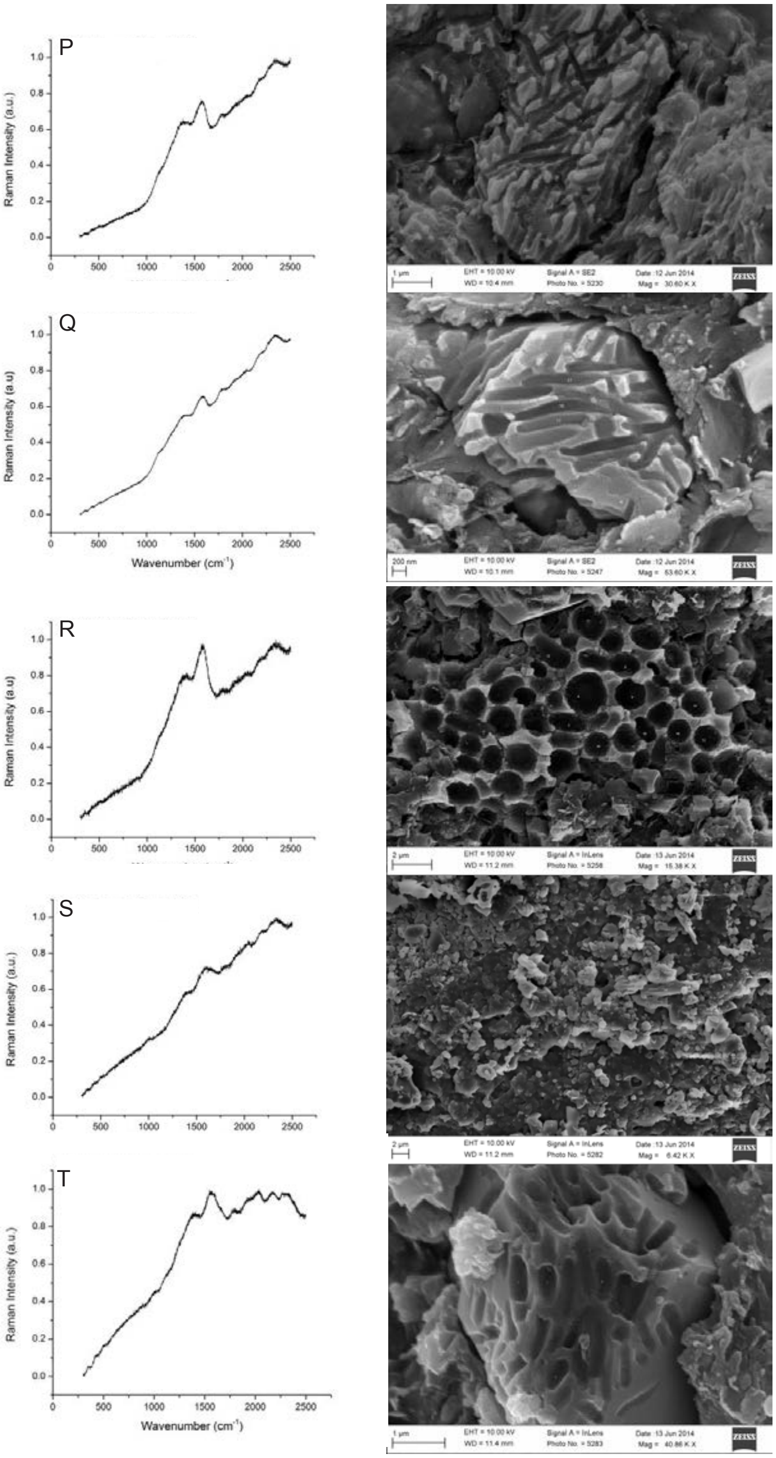
P
Q
R
S
T
Raman Intensity (a.u.)
Wavenumber (cm^{-1})
0
500
1000
1500
2000
2500
0.0
0.2
0.4
0.6
0.8
1.0

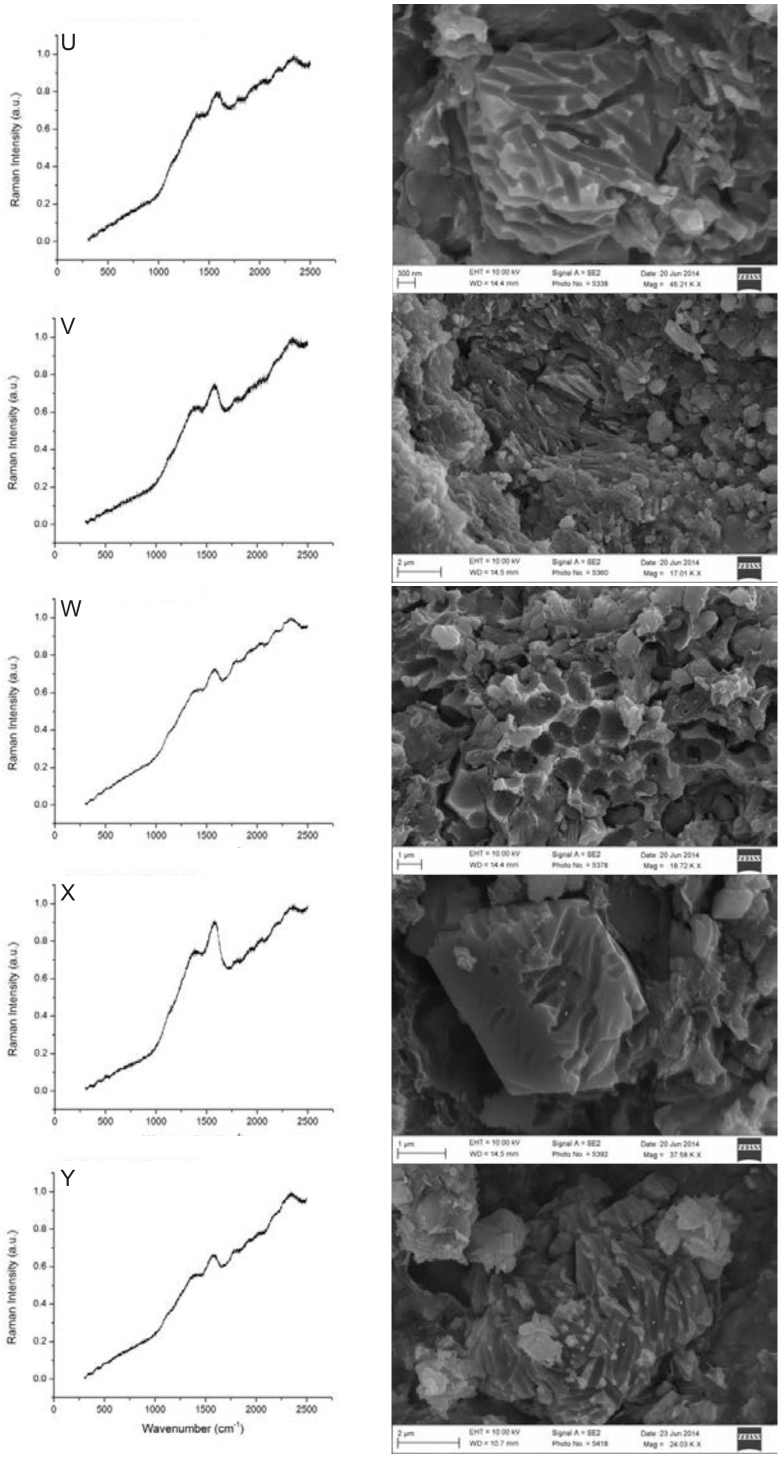
U
V
W
X
Y
Raman Intensity (a.u.)
Wavenumber (cm^{-1})

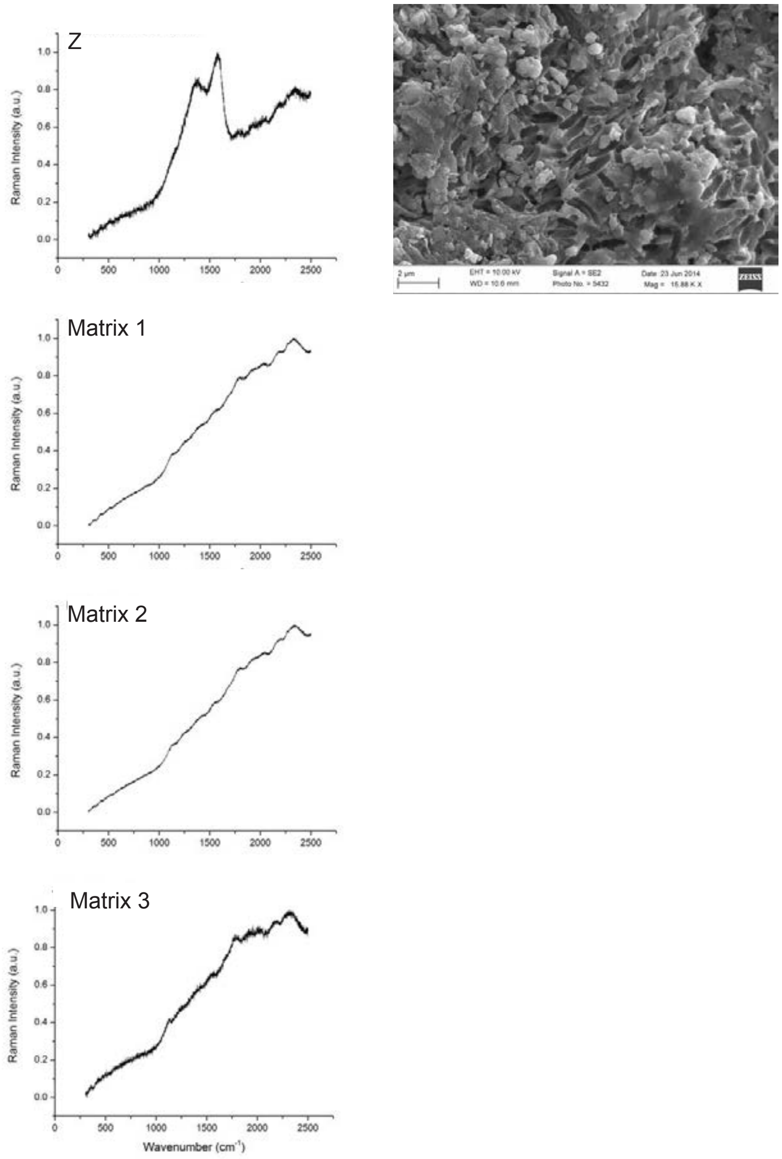

Fig. S4 Raman spectra (left) and corresponding SEM micrograph of samples from specimen CUGB 1401. Sample codes correspond to those in Fig. S2.

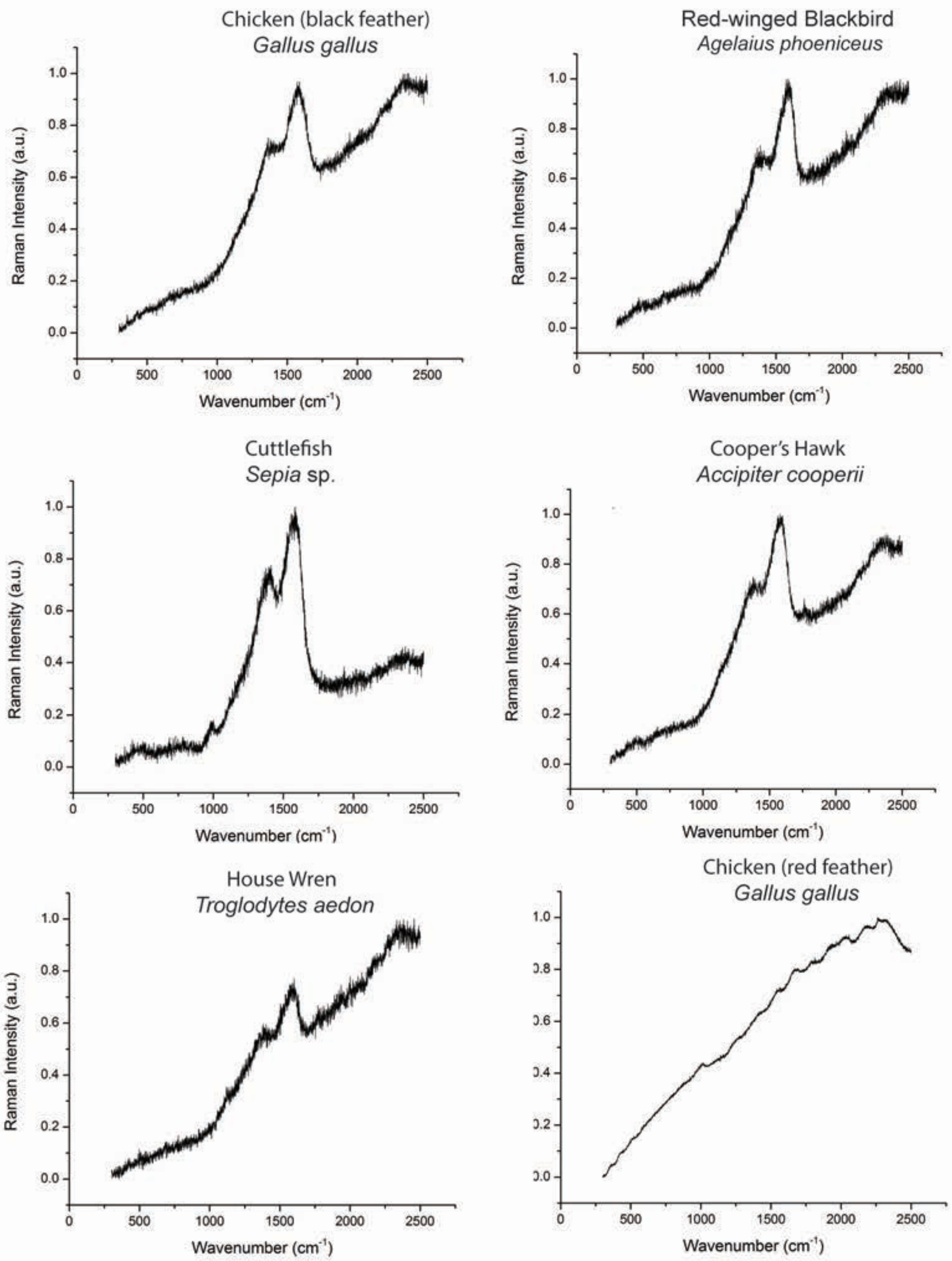
Chicken (black feather)
Gallus gallus
Red-winged Blackbird
Agelaius phoeniceus
Cuttlefish
Sepia sp.
Cooper's Hawk
Accipiter cooperii
House Wren
Troglodytes aedon
Chicken (red feather)
Gallus gallus
Raman Intensity (a.u.)
Wavenumber (cm-1)
0.0
0.2
0.4
0.6
0.8
1.0
0
500
1000
1500
2000
2500

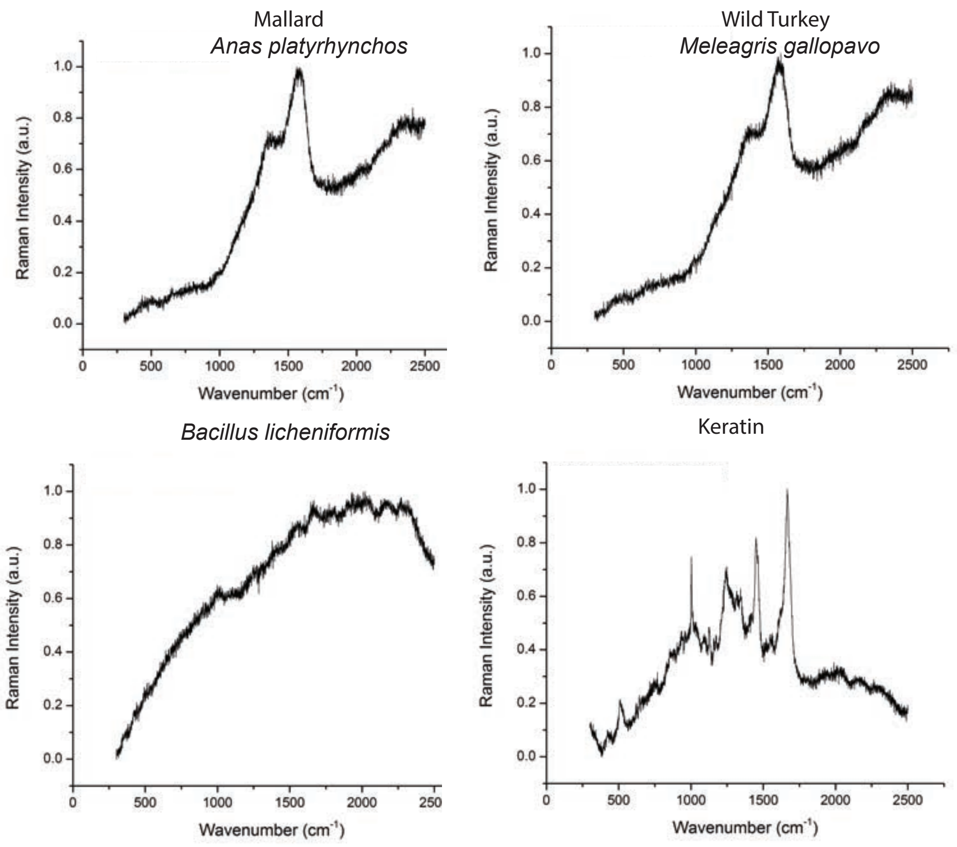

Fig. S5. Raman spectra of eumelanin extracted from black chicken, black red-winged blackbird, brown Cooper's hawk, brown house wren, iridescent mallard, and iridescent wild turkey feathers, a *Sepia* eumelanin standard, melanin extracted from a Rhode Island red rooster feather composed of a high concentration of pheomelanin, the bacterium *Bacillus licheniformis* grown on a white (pigmentless) feather substrate, and keratin from a white sulphur-crested cockatoo feather.

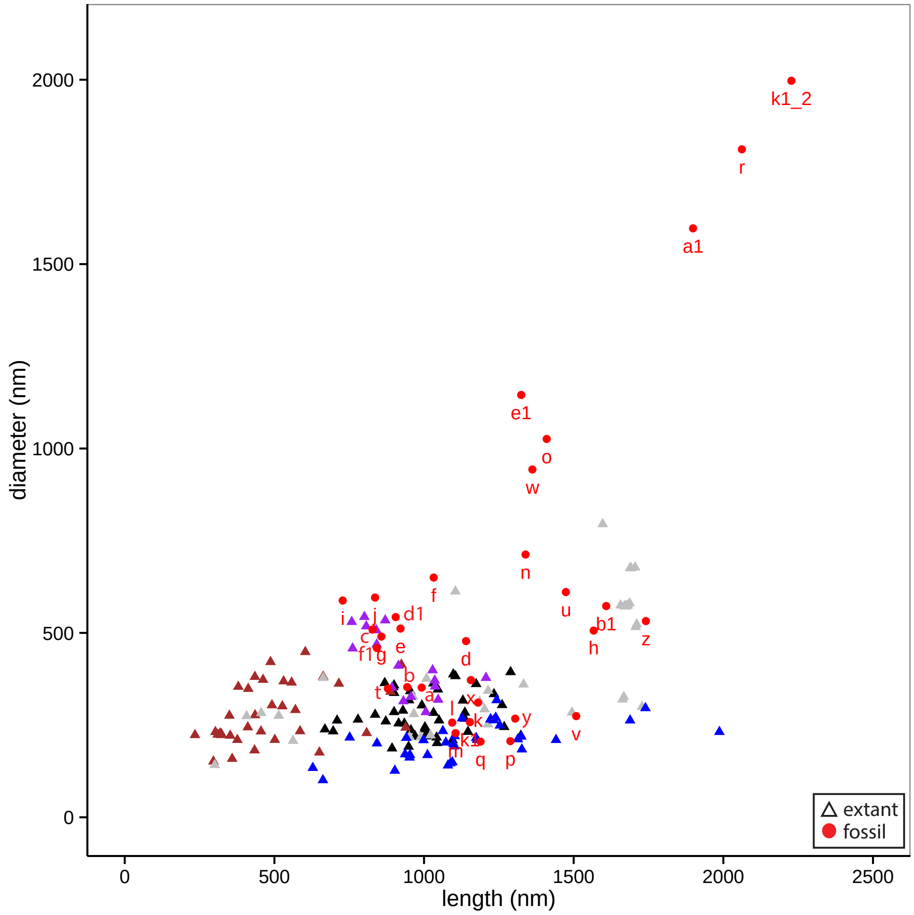

Fig. S6. Diameter vs length for extant avian melanosomes and as measured from CUGB 1401. Colour of triangles (extant samples) corresponds to feather colour. Black, grey, red-brown, blue, and purple-colored triangles represent black, grey, red-brown, penguin-type, and iridescent feathers, respectively. Sample codes correspond to Fig. S1.

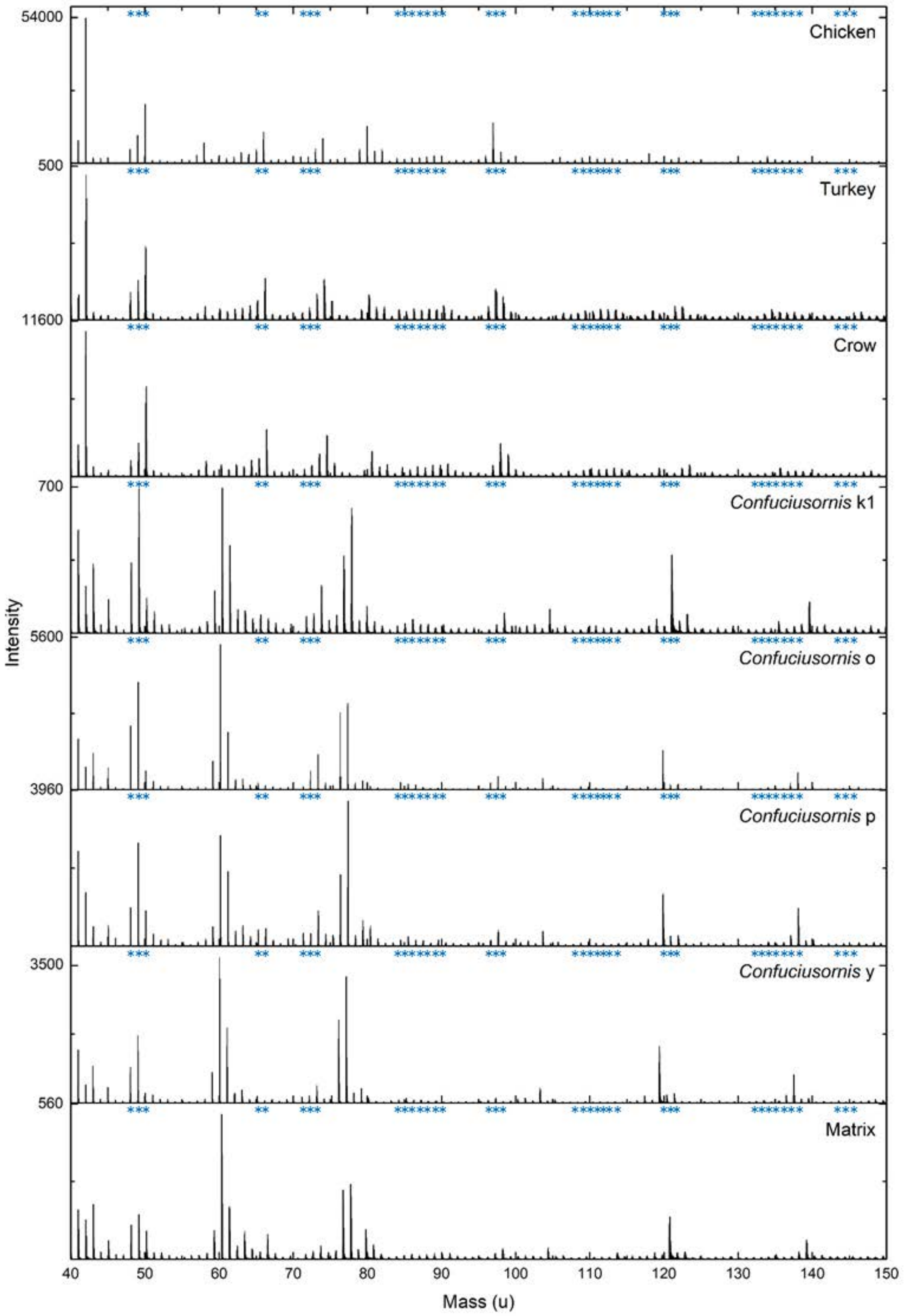

Fig. S7. ToF-SIMS results for three modern melanin samples (chicken, crow, and turkey), CUGB P1401 samples (o, p, y, k1), and the CUGB P1401 matrix. Blue asterisks indicate theoretical masses for eumelanin.

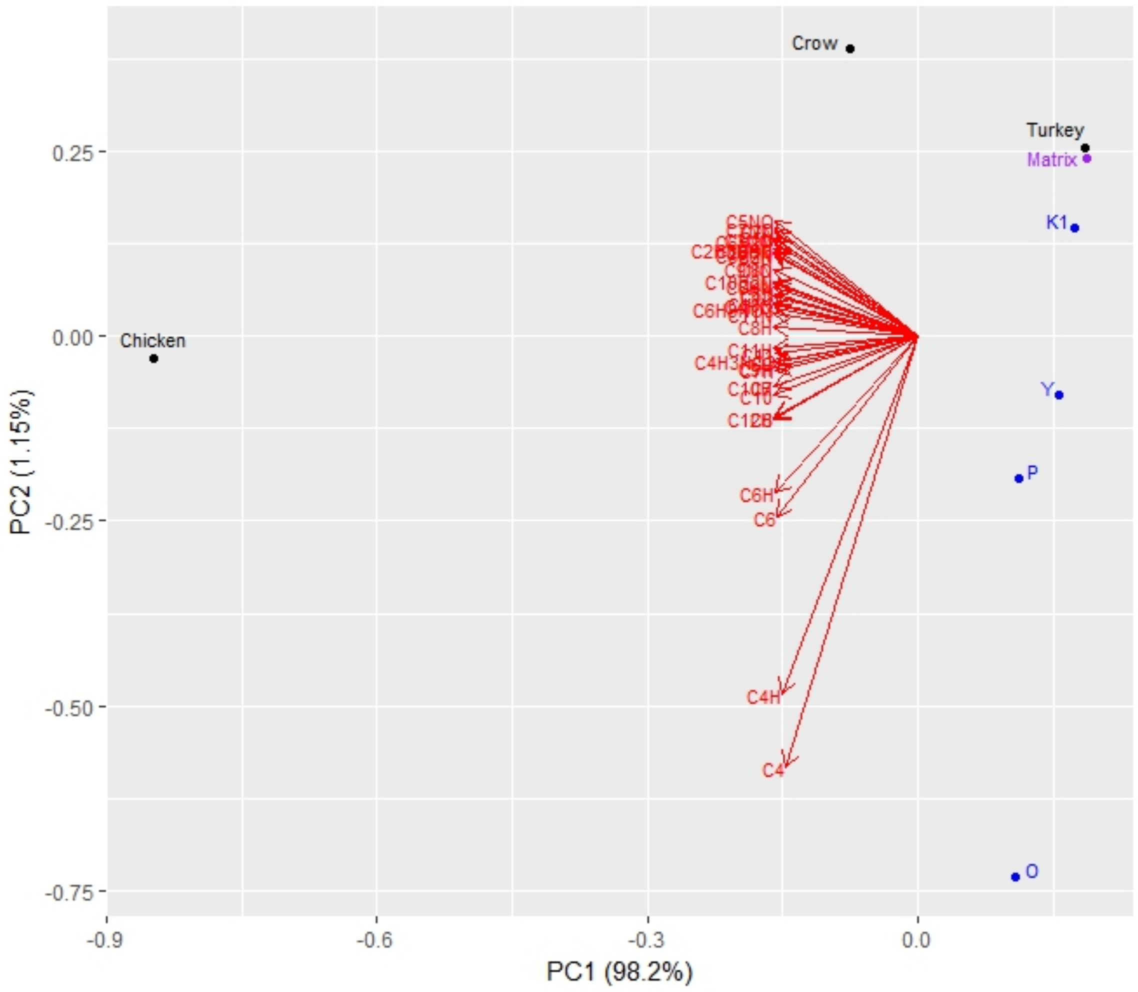

Fig. S8. Principal component plot of ToF-SIMS results from three modern feather samples (black), four CUGB P1401 fossil samples (blue), and one matrix sample (purple). Loadings in red.

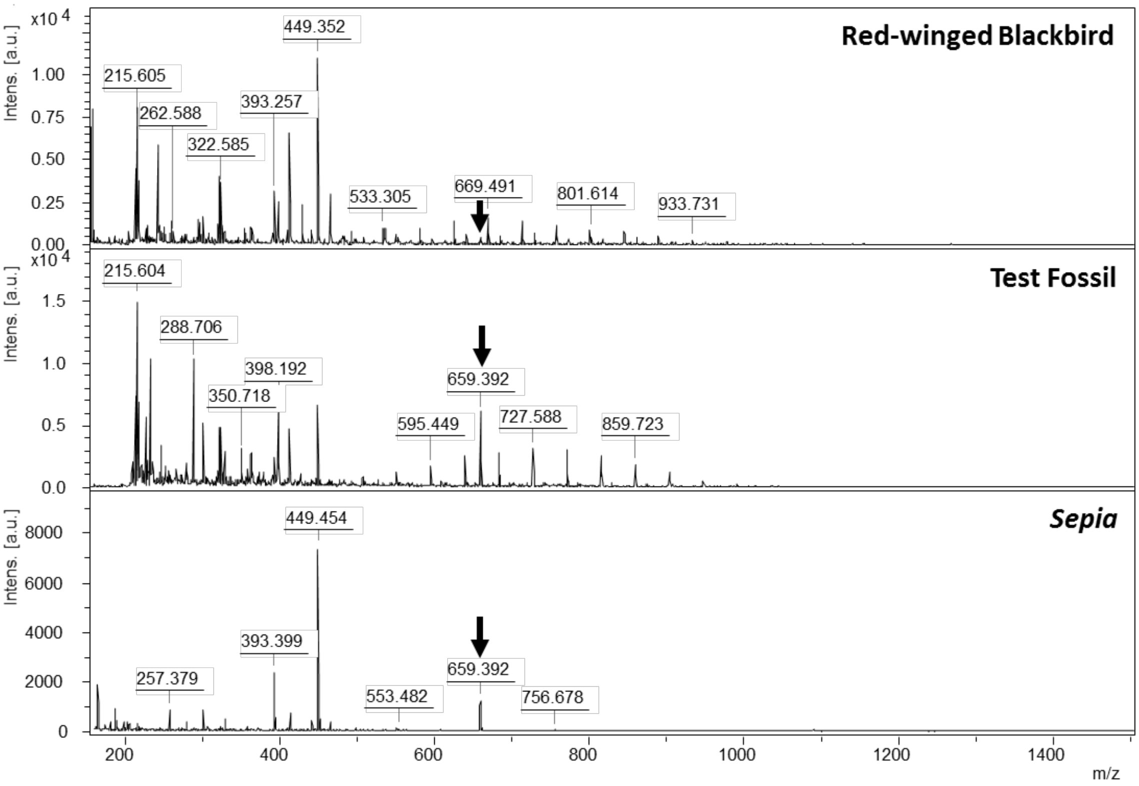

Fig. S9. MALDI results for modern (extracted red-winged blackbird feather melanin and *Sepia* ink) and fossil samples (test fossil from the Yanliao Biota) showing a peak for a melanin dimer ([melanin dimer + Na]$^+$) at approximately 659.4 m/z (arrow).

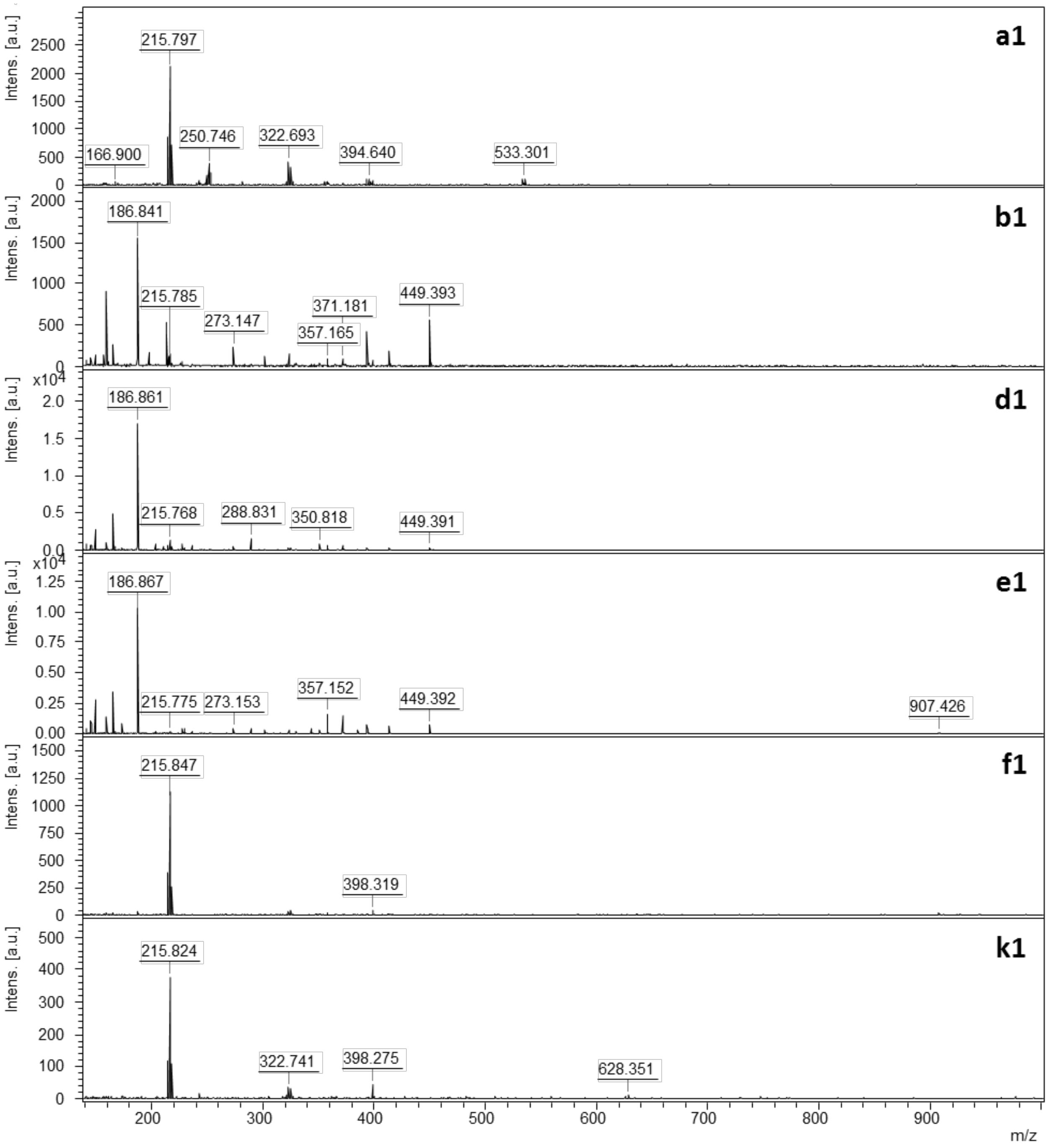
a1
215.797
166.900
250.746
322.693
394.640
533.301
b1
186.841
215.785
273.147
371.181
357.165
449.393
d1
186.861
215.768
288.831
350.818
449.391
e1
186.867
215.775
273.153
357.152
449.392
907.426
f1
215.847
398.319
k1
215.824
322.741
398.275
628.351
Intens. [a.u.]
x10^4
m/z

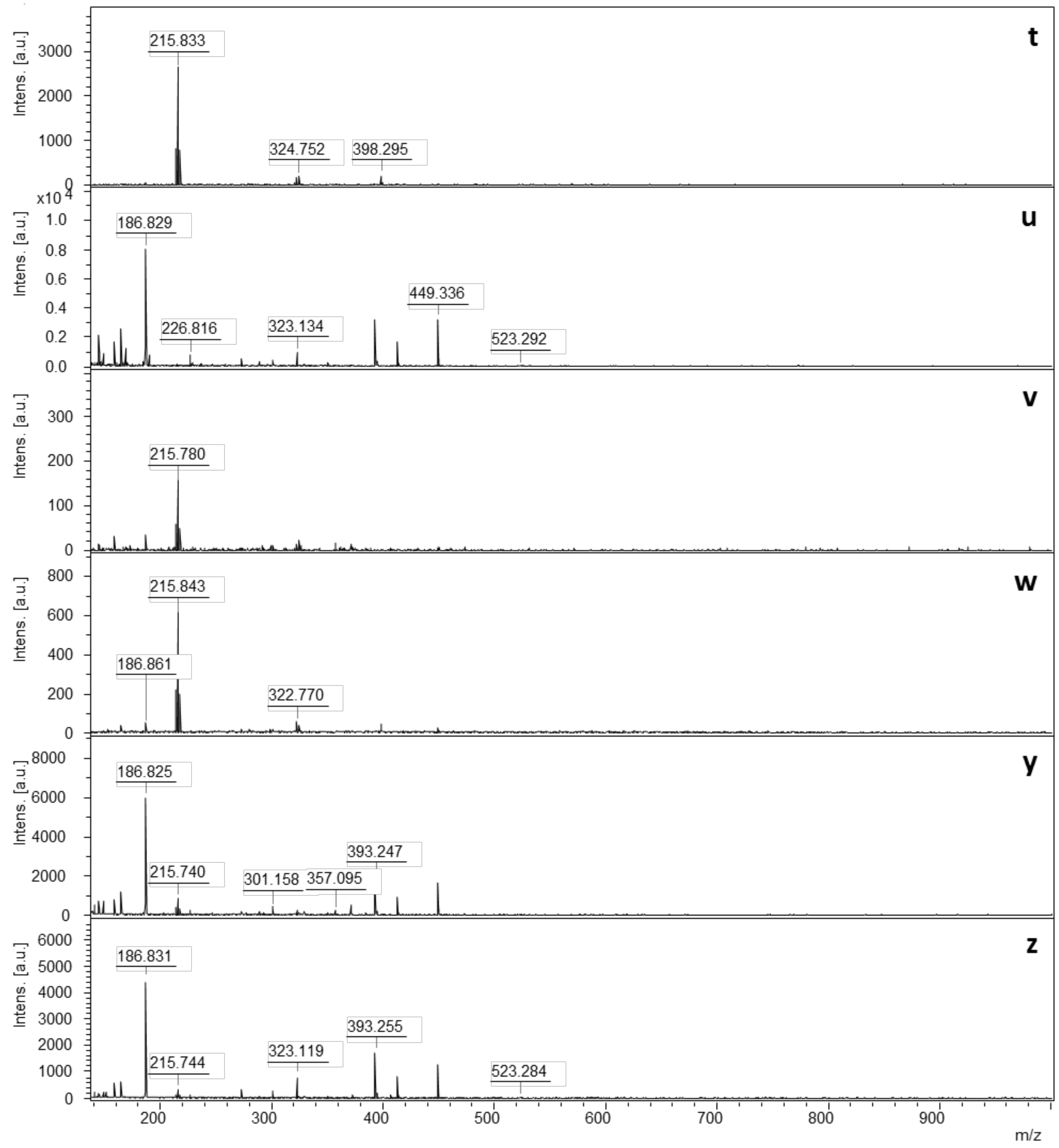

Fig. S10. MALDI results for CUGB P1401 samples, all of which lack the melanin dimer peak visible in the extant melanin and test fossil samples.

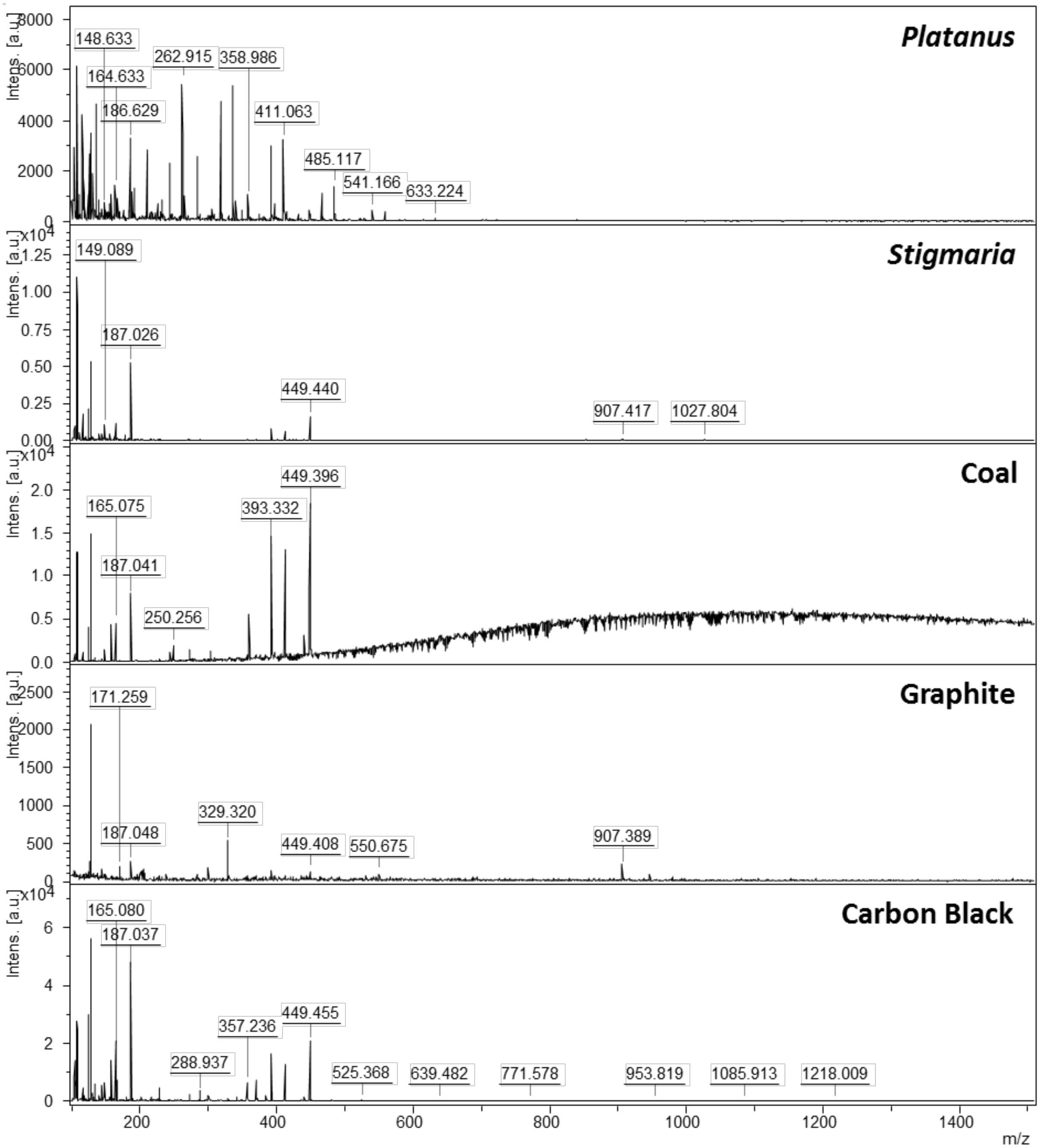

Fig. S11. MALDI results for carbon controls, including two plant fossils (*Platanus* PTRM #20639 and *Stigmaria* CMNH P-21773), bituminous coal, graphite, and carbon black. Carbon controls do not present the melanin dimer peak at approximately 659.4 m/z present in modern melanin samples or the fossil test sample.

Sample number	Location	Eumelanin signal	Melanosome morphology
A	Distal part of feather near bill	No sample	mixed
B	Distal part of cranial feather	Y	mixed
C	Proximal part of cranial feather	N	mixed
D	Dorsocranial feather	Y	mixed
E	Proximal cranial bristle	Y	mixed
F	Distal neck bristle	Y	mixed
G	Distal neck bristle	Y	rod-like
H	Lower neck feathers	Y	rod-like
I	Dark region of spots on front neck feathers	Y	spherical-arranged hexagonally
J	Light region of spots on front neck feathers	Y	spherical-arranged hexagonally
K	Leading edge wing feathers or coverts?	N	rod-like
L	Outer edge of wing, ~midpoint of primary	N	rod-like
M	Outer edge of wing, distal primary	Y	rod-like
N	3rd?primary distal	Y	rod-like
O	Inner primary distal	N	mixed-large
P	Innermost primary distal	Y	rod-like
Q	Innermost primary tip	Y	rod-like
R	Tip of tail feathers	Y	spherical-large
S	Tail or tibial feathers	Y	absent
T	Secondary distal	Y	rod-like
U	Dark spot on secondary	Y	mixed-large
V	Light spot on secondary	Y	rod-like
W	Secondary	Y	spherical-large
X	Secondary middle	Y	mixed
Y	Secondary tip	Y	rod-like
Z	Tibial or tail feather	Y	rod-like
A1	Tail feather	Y	spherical-large
B1	Leg feather	Y	rod-like
D1	Nares feather (counterpart)	N	mixed
E1	Distal tip of head bristle feather (counterpart)	Y	spherical-large, some square-shaped
F1	Back of eye (counterpart)	Y	mixed
K1	Tip of secondaries	Y	spherical-large
Rock1	Non-original matrix	N	absent
Rock2	Original matrix	N	absent
Rock3	Original matrix	N	absent

Table S2. Sampling locations and summary of Raman and morphological data for CUGB 1401.

Sample	Color	Raman Peaks (cm/-1)		
		1	2	3
Chicken	Black	-	1378.52	1578.80
Red-winged Blackbird	Black	1194.45	1385.90	1584.22
Mallard	Iridescent	1166.35	1372.28	1576.94
Wild Turkey	Iridescent	1157.54	1374.42	1577.92
Cooper's Hawk	Brown	1166.51	1373.92	1577.06
House Wren	Brown	1128.57	1384.21	1574.98
A	Fossil	No Sample		
B	Fossil	-	-	-
C	Fossil	-	-	-
D	Fossil	-	1368.38	1575.71
E	Fossil	1149.62	1357.23	1575.49
F	Fossil	-	1372.38	1580.49
G	Fossil	-	1354.34	1573.10
H	Fossil	1135.89	1364.18	1583.02
I	Fossil	1147.05	1362.10	1574.53
J	Fossil	1132.07	1365.38	1574.29
K	Fossil	-	-	-
L	Fossil	-	-	-
M	Fossil	1138.40	1367.29	1576.07
N	Fossil	-	1361.92	1574.17
O	Fossil	-	-	-
P	Fossil	1150.43	1364.90	1570.20
Q	Fossil	1123.83	1364.21	1568.04
R	Fossil	1132.76	1378.29	1583.72
S	Fossil	-	1371.31	1584.06
T	Fossil	1106.39	1374.67	1554.49
U	Fossil	1138.80	1370.15	1568.64
V	Fossil	1152.60	1362.12	1570.17
W	Fossil	1144.59	1360.22	1569.70
X	Fossil	1146.12	1369.35	1577.03
Y	Fossil	1124.02	1350.32	1569.97
Z	Fossil	1133.48	1373.70	1580.39
A1	Fossil	1126.76	1352.73	1574.91
B1	Fossil	1135.55	1357.25	1571.28
D1	Fossil	-	-	-
E1	Fossil	1136.49	1356.50	1569.69
F1	Fossil	-	-	-
K1	Fossil	1159.31	1367.02	1571.37
66	Fossil	1119.74	1375.17	1579.00
67	Fossil	1128.13	1362.39	1572.59

Table S3. Peak fitting table for Raman spectra of extant melanin samples and for samples from CUGB 1401.

Sample	Color	Raman Peaks (cm/-1)							
Feather Keratin	White	514.124	1003.42	1127.33	1146.01	1206.87	1244.22	1454.63	1665.68
Bacillus licheniformis	Bacteria	1006.05	1543.03	1665.28	2028.89	2169.47	2302.65		
Carbon black	Black	1360.57	1590.91						
Coal	Black	1363.00	1587.50						
Graphite	Grey	1354.10	1583.70						
Platanus	Black	1151.30	1351.69	1578.68					
Stigmaria	Black	1369.49	1584.15						

Table S4. Peak fitting table for Raman spectra of feather keratin, the bacterium *Bacillus licheniformis*, and carbon controls. All peak locations were previously reported (Peteya et al., 2017), except those for bituminous coal and graphite.

SUPPLEMENTARY REFERENCES

Burtt EHJ, Ichida JM. 1999. Occurrence of feather-degrading bacilli in the plumage of birds. *The Auk* 116:364-372.

Chiappe LM, Ji S, Ji Q. 2007. Juvenile birds from the Early Cretaceous of China: implications for enantiornithine ontogeny. *American Museum Novitates* 3594:1-46.

Chiappe LM, Ji S, Ji Q, Norell M. 1999 Anatomy and systematics of the Confuciusornithidae (Theropoda: Aves) from the Late Mesozoic of Northeastern China. *Bulletin of the American Museum of Natural History* 242:1-89.

Chiappe LM Marugán-Lobón, J, Zhou Z. 2008. Life history of a basal bird: morphometrics of the Early Cretaceous *Confuciusornis*. *Biology Letters* 4:719-723.

Clarke JA, Ksepka DT, Salas-Gismondi R, Altamirano AJ, Shawkey MD, D'Alba L, Vinther J, Devries TJ, Baby P. 2010. Fossil evidence for evolution of the shape and color of penguin feathers. *Science* 330:954-957.

Hou LH. 2002. *Mesozoic Birds from Western Liaoning in China.* Shenyang: Liaoning Science and Technology Publishing House.

Ji Q, Chiappe LM, Ji S. 1999. A new Late Mesozoic confuciusornithid bird from China. *Journal of Vertebrate Paleontology* 19:1-7.

Galván I, Jorge A. 2015. Dispersive Raman spectroscopy allows the identification and quantification of melanin types. *Ecology and Evolution* 5:1425-1431.

Galván I, Jorge A, García-Gil M. 2017. Pheomelanin molecular vibration is associated with mitochondrial ROS production in melanocytes and systemic oxidative stress and damage. *Integrative Biology* 9:751-761.

Galván I, Jorge A, Ito K, Tabuchi K, Solano F, Wakamatsu K. 2013. Raman spectroscopy as a non-invasive technique for the quantification of melanins in feathers and hairs. *Pigment Cell & Melanoma Research* 26:917-923.

Li L, Wang J, Hou S. 2010a. A new species of *Confuciusornis* from Lower Cretaceous of Jianchung, Liaoning, China. *Global Geology* 29:183-187.

Li Q, Gao KQ, Meng Q, Clarke JA, Shawkey MD, D'Alba L, Pei R, Ellison M, Norell MA, Vinther J. 2012. Reconstruction of Microraptor and the evolution of iridescent plumage. *Science* 335:1215-1219.

Li Q, Gao KQ, Vinther J, Shawkey MD, Clarke JA, D'Alba L, Meng Q, Briggs DEG, Prum RO. 2010b. Plumage color patterns of an extinct dinosaur. *Science*

327:1369-1372.

Lindgren J, Moyer A, Schweitzer MH, Sjövall P, Uvdal P, Nilsson DE, Heimdal J, Engdahl A, Gren JA, Schultz BP, Kear BP. 2015a. Interpreting melanin-based coloration through deep time: a critical review. *Proceedings of the Royal Society B* 282. DOI:10.1098/rspb.2015.0614

Lindgren J, Sjövall P, Carney RM, Cincotta A, Uvdal P, Hutcheson SW, Gustafsson O, Lefèvre U, Escuillié F, Heimdal J, Engdahl A, Gren JA, Kear BP, Wakamatsu K, Yans J, Godefroit P. 2015b. Molecular composition and ultrastructure of Jurassic paravian feathers. *Scientific Reports* 5. DOI:10:1038/srep13520

Lindgren J, Sjövall P, Carney RM, Uvdal P, Gren JA, Dyke G, Schultz BP, Shawkey MD, Barnes KR, Polcyn MJ. 2014. Skin pigmentation provides evidence of convergent melanism in extinct marine reptiles. *Nature* 506:484-488.

Liu SY, Shawkey MD, Parkinson D, Troy TP, Ahmed M. 2014. Elucidation of the chemical composition of avian melanin. *RSC Advances* 4:40396-40399.

Liu Y, Hong L, Wakamatsu K, Ito S, Adhyaru B, Cheng CY, Bowers CR, Simon JD 2005. Comparison of structural and chemical properties of black and red human hair melanosomes. *Photochemical & Photobiological Sciences* 81:135-144.

Marugán-Lobón J, Chiappe LM, Ji S, Zhou Z, Chunling G, Hu D, Meng Q. 2011. Quantitative patterns of morphological variation in the appendicular skeleton of the Early Cretaceous bird *Confuciusornis*. *Journal of Systematic Palaeontology* 9:91-101.

McGraw KJ, Wakamatsu K. 2004. Melanin basis of ornamental feather colors in male Zebra Finches. *Condor* 106:686-690.

McNamara ME, Briggs DEG, Orr PJ, Field DJ, Wang Z. 2013. Experimental maturation of feathers: implications for reconstructions of fossil feather colour. *Biology Letters* 9. DOI:10.1098/rsbl.2013.0184

Moyer AE, Zheng W, Johnson EA, Lamanna MC, Li D, Lacovara KJ, Schweitzer MH. 2014. Melanosomes or microbes: testing an alternative hypothesis for the origin of microbodies in fossil feathers. *Scientific Reports* 4. DOI:10.1038/srep04233

Peteya JA, Clarke JA, Li Q, Gao KQ, Shawkey MD. 2017. The plumage and colouration of an enantiornithine bird from the Early Cretaceous of China. *Palaeontology* 60:55-71.

Smyth JR, Porter JW, Bohren BB. 1951 A study of pigments from red, brown, and buff feathers and hair. *Physiological Zoology* 24:205-216.

Vinther J. 2015. A guide to the field of palaeo colour. *Bioessays* 37:643-656.

Zhang F, Zhou Z, Benton MJ. 2008. A primitive confuciusornithid bird from China and its implications for early avian flight. *Science in China Series D: Earth Sciences* 51:625-639.

Zhang Z, Gao C, Meng Q, Liu J, Hou L, Zheng G. 2009 Diversification in an Early Cretaceous avian genus: evidence from a new species of *Confuciusornis* from China. *Journal of Ornithology* 150:783-790.

Zheng XT 2009. *The Origin of Birds*. Shangdong:Shandong Science and Technology Press.
